# Developmental pathways linked to the vulnerability of adult midbrain dopaminergic neurons to neurodegeneration

**DOI:** 10.3389/fnmol.2022.1071731

**Published:** 2022-12-22

**Authors:** Nilima Prakash

**Affiliations:** Laboratory of Applied Genetics and Stem Cell Biology, Department Hamm 2, Hamm-Lippstadt University of Applied Sciences, Hamm, Germany

**Keywords:** dopamine, mesencephalon, maintenance, survival, neuroprotection, Parkinson’s disease

## Abstract

The degeneration of dopaminergic and other neurons in the aging brain is considered a process starting well beyond the infantile and juvenile period. In contrast to other dopamine-associated neuropsychiatric disorders, such as schizophrenia and drug addiction, typically diagnosed during adolescence or young adulthood and, thus, thought to be rooted in the developing brain, Parkinson’s Disease (PD) is rarely viewed as such. However, evidences have accumulated suggesting that several factors might contribute to an increased vulnerability to death of the dopaminergic neurons at an already very early (developmental) phase in life. Despite the remarkable ability of the brain to compensate such dopamine deficits, the early loss or dysfunction of these neurons might predispose an individual to suffer from PD because the critical threshold of dopamine function will be reached much earlier in life, even if the time-course and strength of naturally occurring and age-dependent dopaminergic cell death is not markedly altered in this individual. Several signaling and transcriptional pathways required for the proper embryonic development of the midbrain dopaminergic neurons, which are the most affected in PD, either continue to be active in the adult mammalian midbrain or are reactivated at the transition to adulthood and under neurotoxic conditions. The persistent activity of these pathways often has neuroprotective functions in adult midbrain dopaminergic neurons, whereas the reactivation of silenced pathways under pathological conditions can promote the survival and even regeneration of these neurons in the lesioned or aging brain. This article summarizes our current knowledge about signaling and transcription factors involved in midbrain dopaminergic neuron development, whose reduced gene dosage or signaling activity are implicated in a lower survival rate of these neurons in the postnatal or aging brain. It also discusses the evidences supporting the neuroprotection of the midbrain dopaminergic system after the external supply or ectopic expression of some of these secreted and nuclear factors in the adult and aging brain. Altogether, the timely monitoring and/or correction of these signaling and transcriptional pathways might be a promising approach to a much earlier diagnosis and/or prevention of PD.

## 1. Introduction

Among the multitude of molecularly, morphologically and functionally diverse cells in the human brain is a quite prominent but numerically very small population, comprising roughly 1% or in average 500,000 of the brain’s nerve cells: the dopamine (DA)-synthesizing neurons in the human ventral midbrain (VM)/brainstem (Pakkenberg et al., 1991). DA is a catecholamine synthesized as a direct derivative of the amino acid tyrosine by the rate-limiting enzyme tyrosine hydroxylase (TH) and the dopa decarboxylase (DDC, or aromatic L-amino acid decarboxylase/AADC) in a common biosynthetic pathway with other catecholamines, such as noradrenaline and adrenaline (Meiser et al., 2013). Because of its binding to a family of at least five different metabotropic DA receptors (DRD1-5) coupled to either stimulatory (G_s_) or inhibitory (G_i_) G-proteins, DA exerts a neuromodulatory function in the brain (Money and Stanwood, 2013; Zhai et al., 2019). In this review, the focus is set exclusively on the midbrain dopaminergic system due to its prominent role in animal and human behavior.

**Figure 1:**
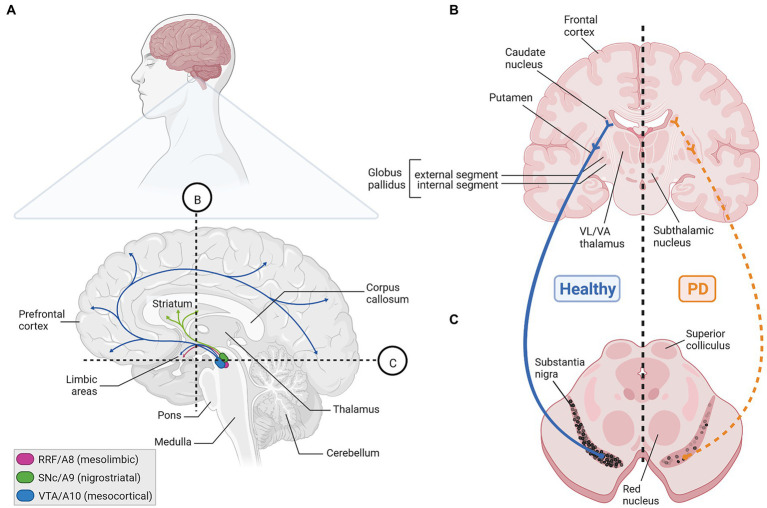
The human mDA system in the adult healthy and PD brain. **(A)** Sagittal view (upper) and enlarged parasagittal section (lower) of the adult human brain, showing the location of the cell bodies (ovals) from the A8 (RRF, pink), A9 (SNc, green), and A10 (VTA, blue) mDA clusters in the brainstem, and their projections (arrows) to the limbic areas (mesolimbic pathway), striatum (nigrostriatal pathway) and PFC (mesocortical pathway). Note that both the VTA and RRF DA neurons project to limbic areas in the mesolimbic pathway. Labeling of other structures (hypothalamus, etc.) and limbic regions, such as the NAc, olfactory tubercle, amygdala, septum, cingulate and perirhinal cortex, in this sagittal view have been omitted for clarity. **(B,C)** Cross-sections at the levels of the dotted lines in **(A)** through the human forebrain **(B)** and midbrain/brainstem **(C)** depicting the location of the projection areas and cell bodies, respectively, of the human SNc DA subset. In the healthy human brain (left side), dark pigmented (neuromelanin-containing) SNc DA neurons extend their axons into the caudate putamen, where they synapse onto their immediate target cells, the striatal GABAergic medium spiny neurons (MSN) and cholinergic and GABAergic fast spiking (FS) interneurons (INs). The MSN neurons project to the internal or external segment of the globus pallidus which, in turn, are connected with the ventrolateral (VL) and ventroanterior (VA) thalamus or subthalamic nucleus in what is known as the direct or indirect pathway, respectively, of the basal ganglia (not depicted here). Retrograde degeneration of the axons and subsequently of the pigmented cell bodies in the SNc (right side) is one key histopathological feature of the PD brain. Created with BioRender.com.

**Figure 2:**
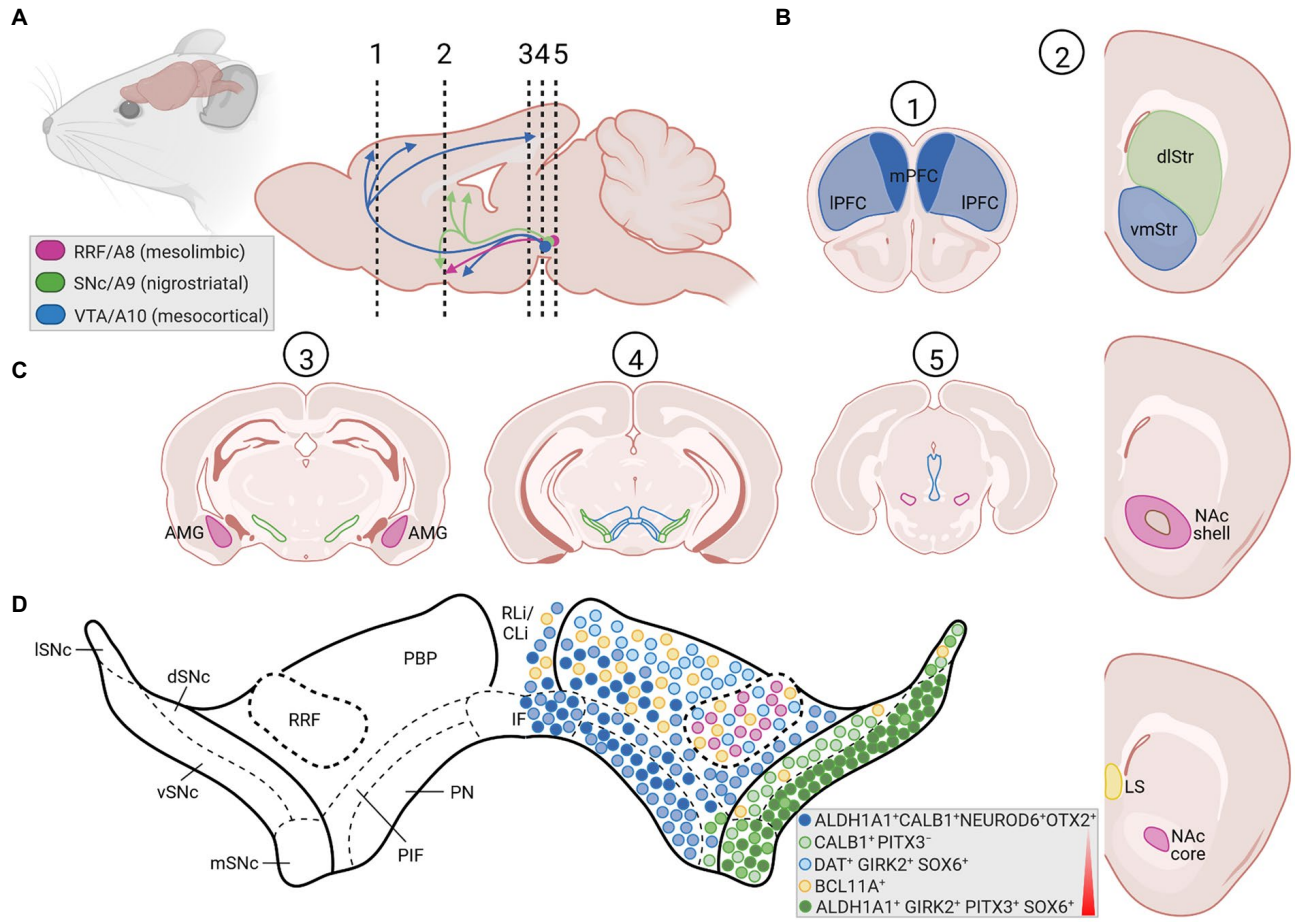
The rodent mDA system in the adult brain. **(A)** Sagittal view (left) and enlarged midsagittal section (right) of the adult rodent (mouse, rat) brain, showing the location of the cell bodies (circles) from the A8 (RRF, pink), A9 (SNc, green), and A10 (VTA, blue) mDA clusters in the VM, and their projections (arrows) to the limbic areas (mesolimbic pathway), striatum (nigrostriatal pathway) and PFC (mesocortical pathway). Note that both the VTA and RRF DA neurons project to limbic areas in the mesolimbic pathway. The dotted and numbered lines indicate the position of the cross sections shown in **(B,C)**. **(B)** Cross-sections of the rodent forebrain at the level of the dotted lines #1 and 2 in **(A)**, depicting the location of the medial (mPFC, dark blue area) and lateral (lPFC, light blue area) PFC (#1) as one target area of the VTA DA subset, of the dorsolateral striatum (dlStr, green area), ventromedial striatum (vmStr, blue area), NAc shell or NAc core (pink area; right panel #2) as preferential target areas of the SNc, VTA and RRF DA subsets, respectively, and of the lateral septum (LS, yellow area) innervated by NEUROD6-and BCL11A-expressing mDA neurons. **(C)** Cross-sections at different rostro-caudal levels of the rodent midbrain, indicated by the dotted lines #3, 4 and 5 in **(A)**, indicating the positions of the SNc (green lines), VTA (blue lines) and RRF (pink lines) DA subpopulations, as well as the location of the amygdala (AMG, pink area) as another target area in the mesolimbic pathway. Labeling of other brain regions in these sagittal and coronal views have been omitted for clarity. **(D)** Enlargement of the mDA region in the medial rodent VM [level of the dotted line and cross-section #4 in **(A,C)**, respectively], showing the SNc (ventral tier, vSNc; dorsal tier, dSNc; medial tier, mSNc; and lateral tier, lSNc), VTA (PBP, parabrachial pigmented nucleus; PN, paranigral nucleus; IF, interfascicular nucleus; PIF, parainterfascicular nucleus; RLi, rostral linear nucleus; CLi, caudal linear nucleus of the raphe), and RRF subdivisions. Colored circles depict the approximate positions and proportions of mDA neurons with distinctive gene expression signatures (legend in bottom right corner) within these subdivisions, as summarized in this article. Colors and shadings indicate the increasing vulnerability (red triangle) of these mDA subsets to neurodegeneration. Intermediate shadings denote mDA subsets not defined here. Created with BioRender.com.

Three major cluster of DA-synthesizing neurons are typically found in the mammalian VM, collectively named midbrain dopaminergic (mDA) neurons. These comprise, in a caudal to rostral numbering, the mDA neurons located in the retrorubral field (RRF, A8 group), substantia nigra pars compacta (SNc, A9 group) and ventral tegmental area (VTA, A10 group; Bjorklund and Dunnett, 2007; Figures 1A, 2A,C). In the rodent brain, the SNc DA neurons or A9 group consist of the dorsal, ventral, medial and lateral tier, whereas the VTA DA neurons or A10 group are made up by the parabrachial pigmented nucleus (PBP), paranigral nucleus (PN), interfascicular nucleus (IF), parainterfascicular nucleus (PIF), and rostral linear nucleus (RLi) and caudal linear nucleus (CLi) of the raphe (Morales and Margolis, 2017; Figures 2C,D). Although this classification is still widely used in the field, extensive analyses of the rodent and human mDA systems revealed that each of these larger mDA groups are made up by molecularly, morphologically and functionally diverse mDA neurons that can be further subdivided into several subgroups, in particular in the SNc and VTA (Roeper, 2013; Poulin et al., 2020). The cell bodies of the SNc DA neurons send their axons primarily to the dorsolateral striatum in rodents (caudate-putamen in primates) within the so-called nigrostriatal pathway, which is a part of the basal ganglia circuitry encompassing also the globus pallidus (internal and external segment) and subthalamic nucleus (Figures 1B,C, 2A,B). The release of DA from these neurons modulates the activity of their target cells in the striatum for the control of voluntary movements and motor learning (Arber and Costa, 2022). The VTA and RRF DA neurons innervate the prefrontal cortex (PFC; mesocortical pathway) and limbic regions of the brain (mesolimbic pathway), including the ventromedial striatum (nucleus accumbens, NAc), olfactory tubercle (OT), amygdala (AMG), septum, cingulate, and perirhinal cortex (Figures 1A, 2A,B). Accordingly, DA release from these neurons is implicated in the control and modulation of cognitive, emotive/affective, motivational/salient and rewarding behaviors (Cox and Witten, 2019; Arber and Costa, 2022). Because of these particular functions, the mDA neurons have gained particular attention in the clinical context: the age-dependent and progressive degeneration of the neuromelanin-containing (dark pigmented) vSNc DA neurons in the human brain is considered a hallmark of PD (Figure 1C), whereas the dysregulation of DA release from the VTA DA neurons is implicated in the pathogenesis of severe neuropsychiatric human disorders, including schizophrenia and substance use disorders (SUD). In fact, insights into the functions of the mDA system in the human context have come primarily from the study and treatment of these diseases. The focus of this review is the particular vulnerability of the mDA neurons to degeneration, and thus restricted to their closer examination in the context of PD.

PD is the second most common age-dependent neurodegenerative disorder after Alzheimer’s Disease currently affecting an estimated more than 6 million people worldwide, with an increasing prevalence of 1 to 3% above the age of 60 and 80, respectively (Tysnes and Storstein, 2017; Feigin et al., 2019). The increased life expectancy, particularly in the high and middle income countries, let some authors to call out actions against an expected “PD pandemic” already in 2018 (Dorsey and Bloem, 2018). Due to the ongoing global COVID-19 pandemic, which can also affect directly or indirectly the developing and adult human brain (Iadecola et al., 2020; Shook et al., 2022), it is at present unclear how the actual numbers of PD cases will develop in the future. PD is characterized by three cardinal symptoms: bradykinesia (slowness of movement), rigidity and resting tremor (Tysnes and Storstein, 2017; Balestrino and Schapira, 2020; Blesa et al., 2022). Bradykinesia can progress to hypokinesia/akinesia (partial or complete loss of movement), and other motor and non-motor/prodromal symptoms, such as postural instability, hyposmia, constipation and sleep disorder, typically accompany the disease, thus causing a broad disability in PD patients (Balestrino and Schapira, 2020; Blesa et al., 2022). The motor symptoms of PD usually arise and develop asymmetrically in the patients, and the initial mDA neuropathology spreads to other neurons and regions of the human brain during the course of the disease (Blesa et al., 2022). The cardinal PD symptoms are caused mainly by the lack of DA in the striatum (Figures 1B,C), leading (in a very simplistic view) to the hyperactivation of the indirect basal ganglia pathway that inhibits voluntary motor routines (Charvin et al., 2018; Blesa et al., 2022). The typical PD medication thus aims at restoring the DA supply in the striatum, primarily by the systemic administration of the DA precursor L-3,4-dihydroxyphenylalanine (L-DOPA, synthesized by TH from L-tyrosine). In contrast to DA, L-DOPA crosses the blood–brain-barrier (BBB) and reaches the remaining SNc DA axon terminals in the striatum, where it is metabolized into DA (Connolly and Lang, 2014; Balestrino and Schapira, 2020). Alternative but less frequently employed symptomatic treatments of PD, which also aim at reestablishing the normal activity of the basal ganglia circuitry, include deep brain stimulation and the not yet implemented (in the clinical routine) gene therapy or transplantation of mDA precursors into the diseased striatum (Blits and Petry, 2016; McIntyre and Anderson, 2016; Balestrino and Schapira, 2020; Harris et al., 2020; Parmar et al., 2020). Due to the unclear etiopathology of PD, particularly of the 90%–95% idiopathic (“sporadic,” because no cause is known) cases, disease-preventing, modifying, or halting treatments are still not available for this neurodegenerative disorder (Charvin et al., 2018; Balestrino and Schapira, 2020). The late diagnosis of PD (usually when at least 50% of the SNc DA neurons have already died; Surmeier et al., 2017; Blesa et al., 2022), puts forward an urgent need of early diagnostic biomarkers enabling a timely intervention in the expected future rise of PD cases (Balestrino and Schapira, 2020; Chmielarz and Saarma, 2020; Tönges et al., 2022).

The single major risk factor for PD is age (Collier et al., 2011; Tysnes and Storstein, 2017; Balestrino and Schapira, 2020). The mDA neurodegenerative process, particularly in the late-onset idiopathic cases, is thus thought to begin well beyond the juvenile and young adult age (Collier et al., 2011; Blesa et al., 2022). Several pathogenic mechanisms have been discussed, some of which appear to be shared by different human neurodegenerative diseases (Gan et al., 2018). Among these, the accumulation and defective proteasomal clearance of misfolded proteins, such as alpha-synuclein (SNCA), dysfunctional mitochondria leading to increased oxidative stress, reduced clearance of these and other defective organelles *via* the mitophagic/autophagic-lysosomal pathway (ALP), as well as a dysregulated calcium (Ca^2+^) homeostasis are considered as the main culprit, which may be exacerbated by a local or systemic immune response (Michel et al., 2016; Gan et al., 2018; Johnson et al., 2019; Panicker et al., 2021). These pathogenic impacts, either individually or altogether, ultimately lead to the apoptotic (programmed) cell death of the SNc DA neurons (Erekat, 2018). The precise reasons why SNc DA neurons are more affected in PD than VTA DA neurons, and why these mDA neurons appear to succumb earlier to degeneration compared to other neurons of the human brain, are not yet clear, but have been linked to increased metabolic energy requirements and a higher Ca^2+^ dependency of the SNc DA neurons [section 2 (Surmeier et al., 2017; Surmeier, 2018; Ni and Ernst, 2022) and references therein]. Moreover, these events are triggered by either a genetic predisposition (in around 5%–10% of PD cases) or environmental impacts (including the exposure to pesticides, heavy metals and infectious agents), or both (Johnson et al., 2019). The genetic (familial) forms of PD (fPD) are caused by mutations in PD-associated genes, so-called *PARK* loci (Hernandez et al., 2016; Benson and Huntley, 2019; Vázquez-Vélez and Zoghbi, 2021). Strikingly, *PARK* genes whose mutations lead to early-onset PD symptoms (i.e., motor symptoms appear during the first to third decade of life) are expressed in the mammalian embryo in a ubiquitous pattern that is not restricted to the VM or mDA system, whereas *PARK* genes causing mostly late-onset PD symptoms [i.e., motor symptoms appear from the fifth decade of life onward, thus resembling idiopathic PD (iPD)] when mutated, start to be expressed only slightly later in the target areas (striatum, cortex) of the mDA neurons (Benson and Huntley, 2019). The latter group include the *PARK1*/*4* locus encoding mutant forms of SNCA and the *PARK8* locus encoding leucine rich repeat kinase 2 (LRRK2). Abnormal accumulation and fibril formation of SNCA lead to the appearance of Lewy bodies and neurites in the PD brain as one of its histopathological hallmarks, whereas LRRK2 is a multifaceted protein with as yet ill-defined functions in the brain whose mutant variants are the second most common genetic risk factor for iPD (Benson and Huntley, 2019; Panicker et al., 2021; Vázquez-Vélez and Zoghbi, 2021). Mutant mice for several of these *PARK* genes display measurable alterations in neurogenesis and synaptic plasticity within the corticostriatal circuitry and other DA-modulated brain circuits at early postnatal or young adult ages, which in some cases also lead to detectable behavioral deficits (Le Grand et al., 2015; Benson and Huntley, 2019; Huntley and Benson, 2020). This indicates that mutations in at least some of the *PARK* genes impair the normal development of the mammalian brain, and strongly suggest that the pathogenic process in PD might begin much earlier than anticipated in the prevailing view. A defective mDA neuron development and maintenance resulting in functionally compromised or reduced numbers of SNc DA neurons could be another early (or the earliest) predisposing factor for the progression to PD in the aging individual or under additional adverse environmental and/or genetic conditions. These early mDA deficits are compensated over a significant amount of time by an enhanced DA metabolism or signaling in the remaining cells (Blesa et al., 2022). In the terminology of (Johnson et al., 2019), deficits in mDA and particularly SNc DA neuron development and maintenance might be considered an additional type of “early” facilitator for the emergence of PD, which *per se* is not sufficient to cause this disease.

This article summarizes mostly rodent and eventually primate *in vivo* studies providing strong evidences for the requirement and neuroprotective role of “developmental factors” in the adult mammalian VM and mDA system. Only those secreted signaling factors and nuclear transcription factors (TFs) active in the mDA domain during prenatal/embryonic development and additionally during adulthood are considered as “developmental factors.” The usability of these factors for preventive or therapeutic interventions in the PD brain is also reviewed, but the myriad of *in vitro* studies in this regard are not addressed here. The involvement of these signaling and/or transcriptional cascades in ongoing neurodegenerative processes associated with PD are not discussed as they were recently reviewed by others (Jha et al., 2022). This article does not delve into the mDA ontogenic and PD-associated programmed cell death (apoptosis; Burke, 2004; Savitt et al., 2005; Erekat, 2018; Robinson et al., 2018) or the role of “classical” neurotrophic factors, such as glial cell derived neurotrophic factor (GDNF), brain derived neurotrophic factor (BDNF), cerebral dopamine neurotrophic factor and mesencephalic astrocyte derived neurotrophic factor (Chmielarz and Saarma, 2020), in this context. The reader is referred to the many excellent reviews on these subjects, of which only the most recent ones are cited in this article.

## 2. Developmental mDA neuron deficits as another hit or facilitator in the “multiple hit” or “stochastic acceleration” hypotheses of Parkinson’s disease?

**Figure 3:**
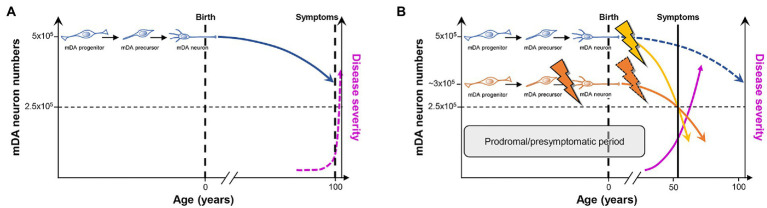
PD as a consequence of “multiple hits” or “stochastic acceleration” of an ongoing SNc DA neuron demise throughout the lifetime of a healthy organism. **(A)** Pre-and perinatal mDA neuron development (indicated by the transition from a proliferating mDA progenitor to a postmitotic mDA precursor to a maturing mDA neuron) endows a healthy human with an average number of 500,000 mDA neurons. Although still debated, a lifelong increased oxidative/nitrosative stress load coupled to a high proteostatic and energetic demand in particularly the SNc DA neurons lead to a continuous demise of some of these neurons (curved blue line) which, however, will not hit the crucial threshold (dashed horizontal black line indicating that only 50% of the mDA neurons remain in the human VM) during the usual human life expectancy. At this threshold, the first PD symptoms would appear (dashed vertical black line), and would worsen with increasing age (dashed pink line). **(B)** As suggested by the “multiple hit” or “stochastic acceleration” hypotheses, the normal mDA neuron demise (dashed blue line) is accelerated by the impact of diverse genetic and/or environmental stressors throughout the postnatal human lifetime (yellow flash and curved line), and will lead to the appearance of the first symptoms and diagnosis of PD (vertical black line) after reaching the critical threshold of mDA neuron numbers (dashed horizontal black line) in the sixth decade of life, as is the case in most (idiopathic) PD cases. Alternatively or in addition, a faulty pre-/perinatal mDA neuron development (orange flash) will render the individual with an already reduced number of mDA neurons at birth, which is still above the critical threshold and can thus be compensated by a heightened DA signaling and metabolism in the remaining mDA neurons from the human VM. Nevertheless, the normal age-related demise of these neurons, most likely together with additional postnatal impacts including a compromised mDA survival (dashed orange flash), will also lead to a much earlier surpassing of the critical threshold and diagnosis of PD (curved orange line). In both cases and due to the current lack of disease-modifying or-halting treatments, PD symptoms and thus severity of the disease will progressively worsen (pink line). The prodromal and presymptomatic period of life in these individuals (gray box) probably represents the best window of opportunity for the modification or, ideally, prevention of PD. Modified after (Le et al., 2009; Collier et al., 2011; Von Linstow et al., 2020).

The idea that a faulty generation, wiring and/or survival, leading to a reduced number of mDA and particularly SNc DA or other neurons already at the earliest stages of life (infancy and youth), might contribute to the emergence of PD and other neurodegenerative disorders in the aging individual is not new and has been proposed by several authors over the last 20 years [(Barzilai and Melamed, 2003; Carvey et al., 2006; Barlow et al., 2007; Ben-Ari, 2008; Le et al., 2009; Von Linstow et al., 2020) and references therein]. This hypothesis was mainly supported by the disentangling of the genetic basis of mDA neuron development in the mammalian embryo (section 3), and the discovery of typically single nucleotide polymorphisms increasing the risk for iPD and fPD that are associated with some of the most prominent genes in this context, such as *EN1* (Fuchs et al., 2009; Haubenberger et al., 2011), *EN2* (Rissling et al., 2009), *NURR1* (*NR4A2*; Xu et al., 2002; Le et al., 2003; Wellenbrock et al., 2003; Zheng et al., 2003; Grimes et al., 2006; Jacobsen et al., 2008; Sleiman et al., 2009), *PITX3* (Fuchs et al., 2009; Bergman et al., 2010; Guo et al., 2011; Haubenberger et al., 2011; Le et al., 2011), *LMX1A*/*B* (Bergman et al., 2009), and *FGF20* (van der Walt et al., 2004; Mizuta et al., 2008; Wang et al., 2008; Pan et al., 2012; Nalls et al., 2019). Most of these point mutations are located in non-coding regions and are thus not expected to impact the function of the corresponding proteins, but rather—if at all—their expression levels (gene dosage; Von Linstow et al., 2020). This suggested that the developmental deficits that might be associated with an increased risk for PD must be subtle and/or masked by compensatory mechanisms during a relevant period of life (Blesa et al., 2022). More recent data indicated that the initiation of *PARK* gene expression in the embryonic rodent brain correlates with the beginning of mDA neurogenesis, and is in some cases strongest in the developing VM (mDA domain) or striatum (mDA target region) [(Sulzer, 2007; Le Grand et al., 2015; Benson and Huntley, 2019) and references therein]. They provided further support for an etiological component of PD that is rooted in early developmental deficits, at least in some cases (Von Linstow et al., 2020), although the precise functions of the corresponding PARK proteins in the healthy brain and especially at these early developmental stages are only beginning to be unraveled (Le Grand et al., 2015; Benson and Huntley, 2019). Nevertheless, PD does not fulfill the criteria of a typical neurodevelopmental disorder because its earliest diagnosis is restricted to very rare cases in a juvenile age (in their teens), and most cases, especially the vast majority of idiopathic cases, are diagnosed only at an advanced age (from their fifties onward; Tysnes and Storstein, 2017). Even if the diagnosis of PD is preceded by a considerable prodromal or pre-symptomatic period of up to 20 years (Blesa et al., 2022), the initiation of the pathogenic process would still be confined to the midlife age in the majority of PD cases (Collier et al., 2011; Von Linstow et al., 2020). A defective development and early survival of the mDA neurons might predispose to the later emergence of PD by acting as an additional risk factor or early “facilitator” for the disease (Figure 3). The term “facilitator” is used in a slightly deviant definition from (Johnson et al., 2019) as a factor that promotes but is not necessary and precedes additional “triggers” to initiate the disease process. This is because there is no human genetic or epidemiologic evidence so far for mutations in developmental genes or deficient developmental processes being a necessary prerequisite for the emergence of PD, and because developmental deficits inherently have to be one of the first if not the first insult in this context. Ultimately, “facilitators,” “triggers,” and “aggravators” have to converge in a probably self-reinforcing process described by the “multiple hit hypothesis” (Carvey et al., 2006; Sulzer, 2007) or, more recently, by the “stochastic acceleration hypothesis” (Collier et al., 2011) to cause the appearance of PD symptoms.

The multiple hit or stochastic acceleration hypotheses essentially suggest that the normal, age-related decline in mDA (particularly SNc DA) neuron numbers in the healthy human brain is precipitated in the PD brain by a combination of different genetic and/or environmental factors (“hits”) impacting on inherent morphologic and/or metabolic properties of the mDA (particularly SNc DA) neurons (Carvey et al., 2006; Sulzer, 2007; Collier et al., 2011). Although the precise numbers of mDA and particularly SNc DA neurons in the adult human brain and, directly related to it, the dimension of an age-related decline in these numbers are still a matter of debate [(Collier et al., 2011; Von Linstow et al., 2020) and references therein], there is a broad consensus that the reduction of mDA neuron numbers in the aging but otherwise unaffected human brain does not reach a critical threshold required to become symptomatic during the usual human life expectancy (Collier et al., 2011; Von Linstow et al., 2020; Figure 3A). In this context, it has been suggested that mDA and in particular SNc DA neurons may have to bear a lifelong additional load (compared with other neuronal cell types) based on their particular morphology and physiology [(Sulzer and Surmeier, 2013; Surmeier et al., 2017; Surmeier, 2018; Hernandez et al., 2019; Ni and Ernst, 2022) and references therein]. Nigrostriatal (SNc) DA neurons possess long and highly branched unmyelinated axons with up to several hundred thousand synaptic sites, posing an extreme proteostatic and energetic (mitochondrial) challenge for their maintenance, and rely on an intrinsic Ca^2+^-based pacemaker activity and low Ca^2+^ buffering capacity, which also impose a high energetic (mitochondrial) demand to maintain a normal Ca^2+^ homeostasis in the cell (Pacelli et al., 2015). Furthermore, all DA neurons are probably exposed to an increased oxidative and nitrative stress load throughout their lifetime, simply because they synthesize and use a chemically very reactive monoamine that can be directly oxidized to quinones and free radicals (Miyazaki and Asanuma, 2008). The time-course of “normal” mDA neuron death under these already stressful but hard if not impossible to manipulate conditions might be precipitated by additional genetic (*PARK* and potentially also “developmental gene” mutations) and/or environmental insults, including pre-or postnatal infections and inflammation as well as environmental toxins, such as the pesticides rotenone and paraquat, the hydroxylated DA analog 6-hydroxydopamine (6-OHDA), or the accidental opioid drug by-product 1-methyl-4-phenyl-1,2,3,6-tetrahydropyridine (MPTP; Carvey et al., 2006; Sulzer, 2007; Collier et al., 2011; Johnson et al., 2019; Figure 3B). These environmental toxins are, in fact, the most widely used agents to induce experimental PD in animal models of this disease, despite the reasonable questioning of this approach and its construct validity for human PD (Dawson et al., 2018). It is particularly a better control of these latter PD risk factors, including the “developmental genes,” which might provide a window of opportunity for the design of preventive measures or at least disease-modifying interventions to ameliorate the complete phenotypic emergence of this so far uncurable neurodegenerative disorder.

## 3. Developmental factors required for mDA neuron survival in the adult brain

**Figure 4:**
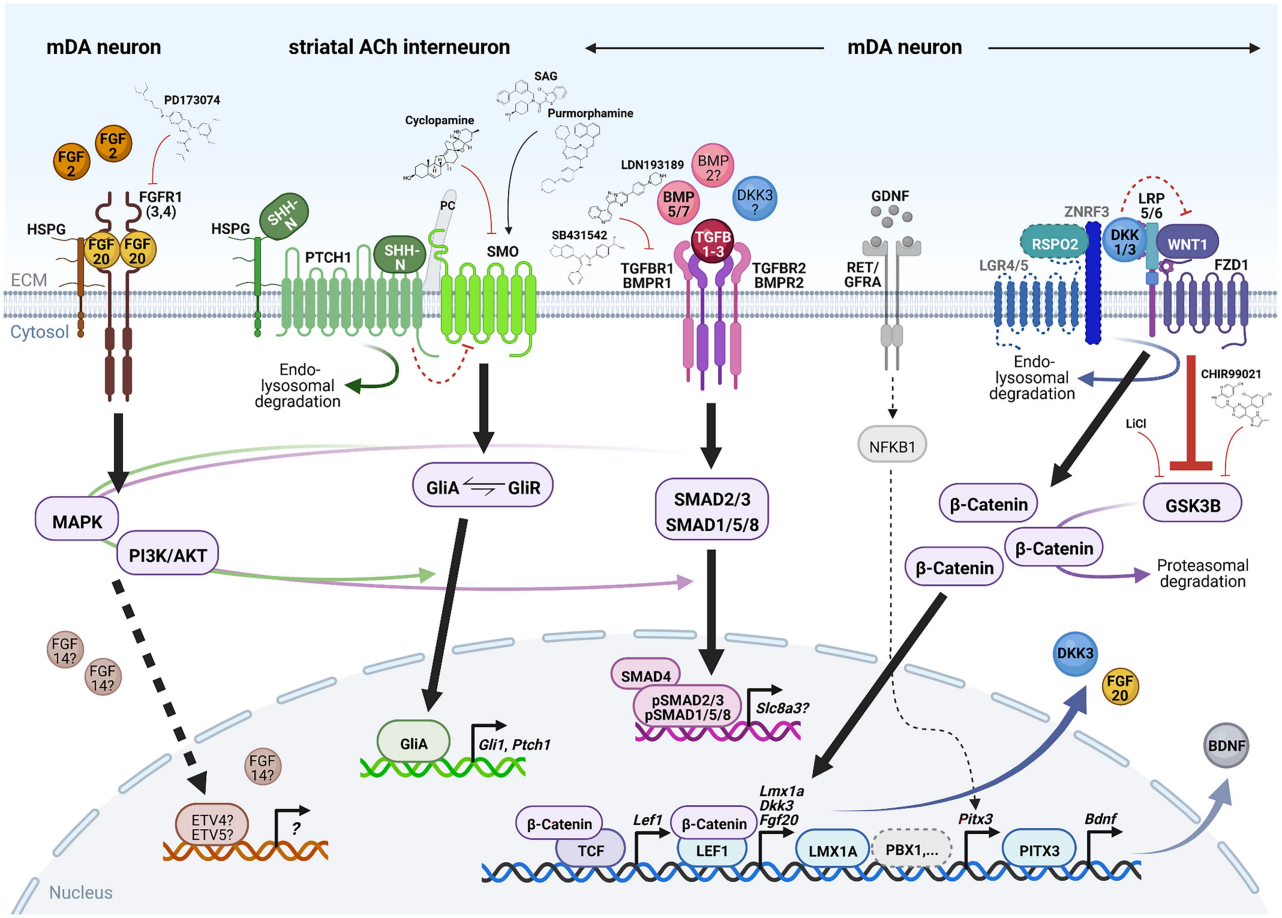
Signaling pathways required for mDA neuron survival in the adult, aging or lesioned mammalian VM. Simplified schematic depiction of the FGF (brown), SHH (green), TGFb/BMP (pink) and WNT/b-catenin (blue) signaling pathways and TFs or transcriptional targets implicated in the adult survival and protection of mDA neurons. FGF, TGFb/BMP and WNT/b-catenin signaling take place in the mDA neurons themselves, whereas SHH signaling is not active in these neurons but in one of their striatal target cells [cholinergic (ACh) INs]. The simplified GDNF/RET/GFRa signaling complex (gray) has been added for clarity in this context. Small molecules frequently employed to activate or inhibit the corresponding pathway are also indicated. Arrows indicate activation, red crossbars indicate inhibition of the corresponding pathway or target gene/protein. Stippled lines label pathways or signaling components not yet demonstrated in adult mDA neurons. Question marks denote still unclear target genes, TFs or ligands for the corresponding pathway during mDA neuron maintenance. See text for details and abbreviations. Created with BioRender.com.

The cellular and molecular underpinnings of mDA neuron development in the rodent and human brain, including the activities of gene regulatory networks and the generation of mDA neuron diversity during this process, are not discussed here and the reader is referred to excellent reviews on these topics (Arenas et al., 2015; Blaess and Ang, 2015; Bodea and Blaess, 2015; Bissonette and Roesch, 2016; Fu et al., 2016; Ásgrímsdóttir and Arenas, 2020; Poulin et al., 2020; Fiorenzano et al., 2021). The development of these neurons proceeds through a series of events in the pre- and postnatal mammalian brain. Prenatal events (during embryonic development) are the initial establishment of a VM domain capable of generating these neurons (regionalization or patterning), the proliferation of already committed mDA progenitors in this domain and their subsequent cell cycle exit to generate postmitotic mDA precursors (induction and fate specification), and the migration of these mDA precursors to their proper positions in the developing VM and at the same time acquirement of their distinctive cellular and molecular phenotypes (differentiation). The maturation of the mDA/basal ganglia system, by establishing the proper connectivity with their efferent targets (Figures 1, 2) and afferent inputs, already begins before birth but continues well into postnatal stages. All these steps are controlled by the (inter-) action of several inter- and intracellular signaling cascades, including the fibroblast growth factor (FGF; Ornitz and Itoh, 2015), sonic hedgehog (SHH; Pak and Segal, 2016; Bangs and Anderson, 2017; Petrov et al., 2017), transforming growth factor beta (TGFb)/bone morphogenetic protein (BMP; Feng and Derynck, 2005; Katagiri and Watabe, 2016), and WNT (“Wingless-type MMTV iNTegration site”; Nusse and Clevers, 2017; Wiese et al., 2018) signaling pathways (section 3.1; Figure 4), and a number of different TFs working up-or downstream of these signaling cascades or other mDA-specific genes (section 3.2; Brodski et al., 2019).

### 3.1. Signaling pathways

#### 3.1.1. FGF signaling

FGFs are a large family of signaling molecules comprising 22 members grouped into seven subfamilies (Ornitz and Itoh, 2015). Five of these seven subfamilies belong to the “canonical” FGFs, including the FGF1, FGF8, and FGF9 subfamilies discussed here, which are secreted into the extracellular space and tightly bound by heparan sulfate proteoglycans (HSPGs) to limit their diffusion and to regulate their interaction with the FGF receptors (FGFRs; Ornitz and Itoh, 2015; Figure 4). The other two FGF subfamilies are either endocrine FGFs (FGF15/19 subfamily, requiring other co-factors for FGFR signaling) or intracellular FGFs (FGF11 subfamily, discussed here, without any obvious extracellular signaling activities; Ornitz and Itoh, 2015). The FGFs bind to four FGFRs (1–4) belonging to the tyrosine kinase receptor superfamily. The FGFRs consist of three extracellular immunoglobulin-like domains and two intracellular tyrosine kinase domains, which are activated after FGF ligand binding and receptor dimerization (Figure 4). The different FGFs exhibit distinct binding affinities to these FGFRs, which are additionally modulated by the existence of splice variants in particularly the extracellular FGFR domains (Ornitz and Itoh, 2015). The activated FGFRs transduce the FGF signal *via* four major intracellular signaling pathways, consisting mostly of sequential phosphorylation/dephosphorylation cascades: RAS/mitogen-activated protein kinase (MAPK), PI3K/AKT serine/threonine kinase, phospholipase C gamma and signal transducer and activator of transcription (Ornitz and Itoh, 2015). Ultimately, the activation of the FGF/FGFR signaling pathway leads to the regulation of gene expression in the nucleus, most prominently *via* the ETS variant TFs ETV4 (PEA3) and ETV5 (ERM; Ornitz and Itoh, 2015; Figure 4).

FGFs have pleiotropic functions during vertebrate development and adult homeostasis, and are thus frequently associated with human disease (Ornitz and Itoh, 2015). The most prominent FGF family members in the context of mDA neuron development and maintenance as well as PD are FGF8, FGF20, FGF2, and FGF14. FGF8, the founding member of the FGF8 subfamily, plays an important role in the early events of mDA neuron generation. These include the initial establishment of the mDA domain in the VM, the induction and proliferation of mDA progenitors in this domain, and their subsequent cell cycle exit and fate specification to generate postmitotic mDA precursors [(Harada et al., 2016; Brodski et al., 2019) and references therein]. However, *Fgf8* is not expressed in the postnatal rodent brain, and the maturation and adult maintenance of the mDA neurons did not appear to depend on FGF8 function (Liu et al., 2021). Thus, other FGFs take over at these later developmental and adult stages and in PD.

The most notable member in this context is FGF20, belonging to the FGF9 subfamily of secreted FGFs (Itoh and Ohta, 2013). FGF20 was initially cloned by homology to the other two FGF9 subfamily members, FGF9 and FGF16, and shown to be highly conserved between rodents and humans (Kirikoshi et al., 2000; Ohmachi et al., 2000; Jeffers et al., 2001). In the mouse embryo, *Fgf20* is widely expressed in ectodermal, mesodermal and endodermal derivatives [(Ornitz and Itoh, 2015) and references therein; (Hajihosseini and Heath, 2002)], but its expression in the developing VM has not been reported so far. Strikingly, *Fgf20* transcription was almost exclusively restricted to the SNc and cerebellum in the adult rodent brain (Ohmachi et al., 2000; Jeffers et al., 2001). Subsequent *in vitro* analyses showed that FGF20 protein was capable of preventing mDA neuron cell death and improving their survival under serum-deprived and excitotoxic/neurotoxic conditions (Ohmachi et al., 2000, 2003; Murase and McKay, 2006). FGF20 bound to the extracellular domain of the c-isoform of FGFR1, which is one out of three (together with FGFR3 and FGFR4) and the most prominently expressed FGFR in adult rodent and human SNc DA neurons (Matsuo et al., 1994; Walker et al., 1998; Sleeman et al., 2012; Boshoff et al., 2018; Figure 4). FGF20 activated the MAPK and PI3K/AKT signaling cascades in these neurons, mediating its neuroprotective effects *in vitro* (Ohmachi et al., 2003; Murase and McKay, 2006; Figure 4). Notably, (Murase and McKay, 2006) also showed that FGF20 signaling *via* the MAPK pathway promotes TH phosphorylation at specific serine residues, thereby enhancing the activity of this enzyme and the synthesis of DA in cultured mDA and particularly SNc DA neurons (Murase and McKay, 2006). More recently and using an immunochemical detection method, (Boshoff et al., 2018) proposed that FGF20 is not expressed in SNc DA neurons but in astrocytes located in the adjacent substantia nigra pars reticulata (SNr), and might thus act in a paracrine manner on these neurons. The exact identity of the cells secreting FGF20 in the adult mammalian VM therefore remains to be established. Nevertheless, signaling *via* the FGFR1 and MAPK/PI3K/AKT pathways was necessary for the sustained survival of mDA neurons in the adult rodent VM, as demonstrated by a reduction of mDA neuron numbers and striatal DA innervation after intranigral viral infection or transfection of a construct encoding a tyrosine kinase-lacking FGFR1 (Corso et al., 2005), in transgenic mice expressing this dominant negative (DN) FGFR1 in catecholaminergic neurons (Klejbor et al., 2006), and after treatment with an FGFR antagonist (Boshoff et al., 2018). The strongest support for a role of FGF20 in adult mDA neuron survival, however, stems from the meanwhile numerous and reconfirmed associations of polymorphisms in the human *FGF20* gene with an increased risk for PD [(Itoh and Ohta, 2013) and references therein; (Nalls et al., 2019)].

FGF2 belongs to the FGF1 subfamily lacking a signal peptide targeting them for secretion, but was nevertheless released into the extracellular space by direct translocation across the cell membrane and was also detected in the nucleus (Ornitz and Itoh, 2015). FGF2 is widely expressed in the developing rodent brain (Giordano et al., 1991), and appeared to be required for the proper establishment of the SNc DA domain during murine development, because zygotic absence (knock-out, KO) or overexpression (OE) of *Fgf2* showed an inverse correlation with the numbers of SNc DA neurons, probably due to overcompensation by other FGFs (Timmer et al., 2007). FGF2 is also expressed in the adult rodent and primate (monkey and human) brain, including the SNc DA neurons and other cells (presumably glia) in the SNr (Bean et al., 1991; Cintra et al., 1991; Tooyama et al., 1994; Gonzalez et al., 1995). Subsequent *in vitro* and *in vivo* analyses corroborated a pro-survival and neuroprotective function of this growth factor in developing and mature mDA neurons [(Liu et al., 2021) and references therein]. Most importantly, the lack of *Fgf2* exacerbated the SNc DA neuron loss after 6-OHDA lesioning of the corresponding null mutant mice, whereas the OE of *Fgf2* in transgenic mice promoted the survival of these neurons under the same conditions (Timmer et al., 2007). However, these findings remain disputed as they were not confirmed by another group using the same *Fgf2^−/−^* KO mice but somewhat different methodological approaches (Zechel et al., 2006). Together with earlier disappointing outcomes of FGF2 infusion into the lesioned primate brain (section 4.1.1), it remains to be seen whether FGF2 may still hold up as an important factor for mDA and particularly SNc DA neuron survival *in vivo* (Liu et al., 2021).

In contrast to the previous FGFs, FGF14 belongs to the FGF11 subfamily of non-secreted, intracellularly localized FGFs that do not appear to interact with FGFRs (Ornitz and Itoh, 2015; Di Re et al., 2017; Figure 4). *Fgf14* was identified as one potential quantitative trait locus associated with an increased number of mDA neurons and increased TH enzyme activity in the VM of inbred mouse strains (Vadasz et al., 2007). In the developing rodent embryo, *Fgf14* transcription is mostly confined to the brain including the VM (Wang et al., 2000; Nouri et al., 2020), but in the adult human and mouse brain, *FGF14*/*Fgf14* mRNA was not or barely detected in SNc DA neurons (Smallwood et al., 1996; Wang et al., 2002). However, *FGF14*/*Fgf14* mRNA and FGF14 fusion proteins were strongly expressed within the basal ganglia circuitry including efferent output and afferent input areas of these neurons, such as the striatum (caudate putamen), globus pallidus and SNr (Wang et al., 2002). *Fgf14* KO mice exhibited dyskinetic movement disturbances, which were not due to changes in mDA neuron numbers and their striatal projections or DA metabolism but due to reduced responses to DA agonists (Wang et al., 2002). This study suggested that FGF14 is implicated in mDA synaptic transmission and plasticity, potentially through its direct or indirect interaction, respectively, with voltage-gated sodium (Na^+^) or potassium (K^+^) and Ca^2+^ channels (Di Re et al., 2017). FGF14 might therefore act as an anterograde or retrograde intracellular modulator of DA neurotransmission in the basal ganglia system crucially involved in motor and other behavioral control (Wang et al., 2002). Several polymorphisms in the human *FGF14* gene have been associated with an increased risk for neuropsychiatric disorders, including DA-related ones such as schizophrenia and SUD [(Di Re et al., 2017) and references therein].

#### 3.1.2. SHH signaling

The SHH signal transduction pathway is probably the most complex and least understood of all four signaling pathways discussed here. SHH is one of three lipid-modified members of the hedgehog (HH) protein family, whose N-terminal bioactive fragments (SHH-N) bind to and activate the 12-pass transmembrane protein Patched 1 (PTCH1; Pak and Segal, 2016; Petrov et al., 2017; Figure 4). Binding of SHH-N to PTCH1 is aided by several co-receptors (CDON, BOC, GAS1) necessary for HH pathway activation (Pak and Segal, 2016; Petrov et al., 2017). In the absence of SHH, PTCH1 represses the activity of the seven-pass transmembrane protein Smoothened (SMO) by a not yet understood mechanism (Figure 4). Binding of the SHH-N ligand to the PTCH1 receptor causes the endocytosis and lysosomal degradation of this ligand-receptor complex and the relocation of SMO from an intracellular compartment to the primary cilium (PC), where PTCH1 is normally located (Figure 4). Subsequently, SMO and the downstream effectors of this pathway, the GLI zinc finger TFs comprising three members in vertebrates (GLI1-3), are activated by a complex sequence of protein kinase-, G-protein-coupled receptor kinase- and phosphatase-mediated phosphorylation and dephosphorylation events (Pak and Segal, 2016; Petrov et al., 2017). *Gli1* is a direct target gene of the HH signaling pathway and thus positive feedback regulator of this pathway, whereas GLI2 and GLI3 function mostly as transcriptional activator (GliA) or repressor (GliR), respectively (Pak and Segal, 2016). Phosphorylation of the full-length GLI proteins (GliA) targets them for proteolytic cleavage and proteasomal degradation, which in the first case yields the GliR form of the corresponding protein. The signaling outcome of the HH pathway is determined by the balance between the GliA and GliR forms of these TFs and their control of the transcriptional output in the nucleus, including positive and negative feedback regulation by their targets *Gli1* and *Ptch1*, respectively (Pak and Segal, 2016; Figure 4). The PC plays a fundamental role in HH signal transduction, as evidenced by the disruption of this pathway in mouse mutants for several structural and functional components of the PC (Bangs and Anderson, 2017). Not only PTCH1 and SMO have to localize to the PC for proper HH signaling but also the GLI TFs, which are bound as full-length (GliA) proteins by SUFU, a negative regulator of this pathway, and translocated into the tip of the PC. In the PC, GliA proteins will either serve as a “reserve pool” for HH signal transduction or become phosphorylated and processed into the GliR form (Pak and Segal, 2016; Bangs and Anderson, 2017). The PC also plays a role in other signal transduction pathways, such as WNT/b-catenin and TGFb signaling (Ma et al., 2022).

SHH is the sole HH protein in the developing and adult mammalian brain, and has therefore been implicated in a variety of neurodevelopmental processes and neurodegenerative diseases (Brodski et al., 2019; Yang et al., 2021). In the developing mouse VM, SHH signaling is required only for the ventral patterning and establishment of the mDA progenitor domain, but not for mDA neuron differentiation. This is due to the temporally limited expression until midgestation of essential SHH signaling components (PTCH1, GLI1/2/3 and SHH itself) in this region [(Brodski et al., 2019) and references therein]. Some components of SHH signaling, such as SMO, continue to be expressed in maturing mDA neurons and control the proper axonal outgrowth of a subset of these neurons (Brodski et al., 2019). The conditional inactivation (cKO) of structural and functional components of the PC in mice also resulted in a partial or transient reduction of mDA progenitors and neurons, depending on the developmental timepoint at which SHH signaling was affected in these cKO mice [(Brodski et al., 2019) and references therein].

Despite the limited role of SHH signaling during mDA neuron development, the improved mDA neuron survival after intrastriatal or supranigral injection of recombinant human (rh) lipid-modified SHH-N protein were among the first reports suggesting a neuroprotective role of this signaling pathway in adult mDA neurons (Dass et al., 2002; Tsuboi and Shults, 2002). Two seminal studies by Gonzalez-Reyes et al. (2012) and Ortega-de San Luis et al. (2018) later demonstrated that SHH is expressed by virtually all mDA neurons (including the SNc) in the adult mouse brain, although essential components of this pathway, such as the PTCH1/2 receptors, were not detected in these neurons. Moreover, *Smo* cKO in dopamine transporter (DAT/SLC6A3)-expressing (maturing) mDA neurons did not result in any overt mDA phenotype up to 18 months of age, even after a neurotoxic (6-OHDA) challenge (Zhou et al., 2016). The hyperactive locomotor phenotype, attenuated psychostimulant response, and reduced *Bdnf* but unchanged *Gdnf* and *Bmp7* transcription in the VM of young adult *Dat*-*Smo* cKO mice observed by Zhou et al. (2016) might thus be due to the known function of SMO as an axonal guidance receptor in mDA neurons (Hammond et al., 2009). *Dat*-*Shh* cKO mice did not display an obvious mDA phenotype at birth, but revealed a progressive 40% loss of in particular the SNc DA neurons starting at 4 months and plateauing in the 8 month of age (Gonzalez-Reyes et al., 2012). These losses were accompanied by highly dynamic changes in striatal mDA innervation and DA release, locomotion as well as in the expression of other proteins involved in DA neurotransmission, such as TH, DAT, DRD2 and the vesicular monoamine transporter VMAT2 (SLC18A2), suggesting temporary compensatory mechanisms in these mouse mutants (Gonzalez-Reyes et al., 2012). As a cautionary tale, the behavioral and gene expression phenotypes of the *Dat*-*Smo* and *Dat*-*Shh* cKO mice might have also been caused, at least in part, by the disruption of one *Dat* allele in the corresponding Cre driver mice (Giros et al., 1996; Zhuang et al., 2005; Backman et al., 2006). The absence of the PTCH1 receptor in adult mDA neurons prompted the investigation of a non-cell autonomous (paracrine) neuroprotective action of SHH within the nigrostriatal pathway. Indeed, mDA neuron death was paralleled by a progressive loss of cholinergic and GABAergic FS INs in the *Dat*-*Shh* cKO striatum (Gonzalez-Reyes et al., 2012). This finding was later refined by (Ortega-de San Luis et al., 2018) using more sophisticated methods, showing that the physiologically induced loss of more than 95% mDA neurons resulted in a selective reduction of only GABAergic FS INs but not cholinergic INs and, conversely, *Smo* cKO in these two striatal IN populations affected the adult survival of only the cholinergic but not the GABAergic FS INs. In search for a non-mDA source of SHH in the striatum, the GABAergic FS and cholinergic INs themselves were identified as potential auto- or paracrine suppliers of this signaling molecule (Ortega-de San Luis et al., 2018). Although Gonzalez-Reyes et al. (2012) proposed that *Gdnf* transcription in these striatal INs, one of the most potent neurotrophic factors for mDA neurons (Chmielarz and Saarma, 2020), was engaged in a mutual negative feedback loop with the transcription of *Shh* in mDA neurons, some of their findings were not reproduced by Ortega-de San Luis et al. (2018). Adult *Smo* cKO mice lacking an active SHH pathway in the cholinergic INs showed a modest but progressive loss of mDA neurons which, however, was not due to changes in striatal GDNF expression. These data revealed that the mDA neurons and part of their striatal target cells are engaged in an intricate homeostatic relationship whose details still remain to be clarified. All three neuronal populations require the trophic support of a factor produced by either one of the other neuronal population or by themselves: striatal cholinergic INs require SHH produced by the mDA neurons or released locally; striatal GABAergic FS INs require mDA input independent of SHH; and mDA neurons require an unknown cholinergic IN-derived trophic factor for their survival.

Striatal cholinergic INs are also thought to play a crucial role in the emergence of L-DOPA induced dyskinesias (LIDs), a severe side-effect of prolonged L-DOPA medication (Shen et al., 2022). The potential role of SHH signaling in the regulation of striatal cholinergic IN physiology prompted (Malave et al., 2021) to test whether the acute or chronic co-application of a SHH agonist (SAG or purmorphamine) or antagonist (cyclopamine) during L-DOPA treatment would attenuate or increase, respectively, the appearance of LIDs in mouse and primate PD models. This was indeed the case and traced back to the inactivation or activation, respectively, of the MAPK pathway downstream of SMO in these INs (Malave et al., 2021; Figure 4). *Smo* cKO in striatal cholinergic INs or *Shh* cKO in young mDA neurons resulted in the progressive worsening of LIDs despite the lack of an mDA phenotype in these mice, whereas the OE of a constitutively active SMO protein in a genetic PD mouse model blocked the appearance and intensification of LIDs despite the severe mDA phenotype of these animals (Malave et al., 2021). In an elegant series of experiments, Malave et al. (2021) showed that the repeated optogenetic stimulation of mDA burst firing led to LID-like behavior intensification in an L-DOPA-independent manner. This was most likely due to the exhaustion of SHH signaling from the mDA neurons to the striatal cholinergic INs, because a single dose of the antagonist cyclopamine prior to stimulation onset exacerbated, whereas the agonist SAG blocked this effect. Importantly, these findings suggest that an imbalance between excessive DA signaling (due to L-DOPA administration) and strongly diminished SHH signaling (due to mDA neurodegeneration) in striatal cholinergic INs leads to the emergence of LIDs. The co-administration of SHH agonists might restore this balance in the L-DOPA treated PD brain and provide an effective or even preventive anti-dyskinetic treatment.

The importance of the PC in nigrostriatal DA neurotransmission and survival has only been recognized recently. PC morphology (length) in the striatal targets (MSN and IN) of the mDA neurons was regulated by DA-mediated (on the presynaptic side) and DRD2-specific (on the postsynaptic side) neurotransmission, leading to elongated PC in their absence (Miyoshi et al., 2014). Elongated PC were also detected in postmortem striatal neurons of iPD patients and unilaterally treated 6-OHDA mice, suggesting the loss of mDA innervation as the causative factor in both cases (Schmidt et al., 2022). PC formation was reduced specifically in striatal cholinergic INs and astrocytes of humanized mice carrying autosomal dominant mutations (R1441G and G2019S) in the human *LRRK2*/*PARK8* gene (Dhekne et al., 2018; Khan et al., 2021). These *LRRK2* mutations are thought to increase the kinase activity of the mutant proteins, and were detected in late-onset fPD (Vázquez-Vélez and Zoghbi, 2021). *Lrrk2* is highly expressed in the adult mouse striatum (Benson and Huntley, 2019). The increased phosphorylation of essential PC components by the mutant LRRK2 proteins, although not directly demonstrated in striatal cells, interfered with PC formation and disrupted SHH signaling in LRRK2 R1441G mouse embryonic fibroblasts and striatal cholinergic INs as well as LRRK2 G2019S human induced pluripotent stem cells (hiPSCs; Dhekne et al., 2018; Khan et al., 2021). This suggested that the survival of striatal cholinergic INs and, even more importantly, the intricate SHH-mediated nigrostriatal homeostatic feedback loop might be compromised in the *LRRK2* mutant PD brain. PC are also present in a substantial proportion of adult SNc DA neurons (non-SHH-responsive, see above). Their enhanced biogenesis and elongation under increased mitochondrial oxidative stress conditions (MPTP lesioning) appeared to exert a neuroprotective effect by augmenting the autophagic removal of dysfunctional mitochondria and suppressing the apoptotic death of these neurons (Bae et al., 2019). These results were partly corroborated by a recent study showing that the cKO of a crucial structural and functional component of the PC in maturing mDA neurons (*Dat*-*Ift88* mice) resulted in the loss of approximately 20% SNc DA neurons and a corresponding decrease of striatal mDA innervation and DA content, as well as an increase of PC length in striatal neurons (Mustafa et al., 2021). The reduction of SNc DA neuron numbers in the *Dat*-*Ift88* cKO mice was not further accentuated by MPTP treatment (Mustafa et al., 2021), in contrast to the report by Bae et al. (2019). The most likely explanation for these discrepancies are methodological differences (constitutive genetic ablation vs. viral OE-mediated knockdown of the PC component, and subchronic vs. acute administration of MPTP) between the two studies (Bae et al., 2019; Mustafa et al., 2021). Mustafa et al. (2021) also proposed that the loss of SNc DA neurons in the *Dat*-*Ift88* cKO mutants affected primarily a PC-bearing subset of these neurons displaying a high intrinsic electrophysiological activity that can be further stimulated by DA, which might therefore be particularly prone to metabolic stress and degeneration. Proliferating neural precursors and maturing mDA neurons derived from iPD and fPD hiPSCs, considered as “rejuvenated” cells representing fetal stages in human development, were recently reported to display mitochondrial respiratory deficits, dysregulated pathways associated with PC and SHH signaling, and shortened PC (Schmidt et al., 2022). These phenotypes were reversed by the application of cyclopamine, a SHH antagonist directly binding to SMO, or the withdrawal of purmorphamine (a SMO agonist; Figure 4) from the culture media, and thus interpreted as a consequence of increased SHH signaling in the iPD- and fPD-derived neural cells (Schmidt et al., 2022). The mitochondrial impairments in these “rejuvenated” cells support the assumption of a developmental onset of metabolic deficits in PD (discussed in section 5), although it remains to be clarified how the proposed SHH signaling impairments in the iPD/fPD mDA precursors and neurons can be reconciled with the absence of the PTCH1/2 receptors and GLI TFs in these cells.

#### 3.1.3. TGFbeta/BMP signaling

Long known as secreted neurotrophic proteins, the 33 members of the TGFb superfamily in mammals are grouped in two major subfamilies based on their sequence and structural homology: the TGFb-like subfamily, comprising the three TGFB1-3 isoforms (discussed here), activins, nodal and some growth and differentiation factors (GDFs); and the BMP-like subfamily, consisting of 13 BMPs (discussed here) and most GDFs (Weiss and Attisano, 2013). The bioactive peptides of this superfamily are cleaved from larger precursor proteins and dimerized by the formation of disulfide bonds. After release, their activity in the extracellular space is controlled by the interactions with the extracellular matrix (ECM) and a number of inhibitors (Weiss and Attisano, 2013). Dimeric TGFb superfamily ligands bind to a heterotetrameric receptor complex on the cell surface, consisting of two type I [or activin receptor-like kinases (ALKs)] and two type II transmembrane serine/threonine kinases (Figure 4). For TGFB1-3 and BMPs, these are mostly the TGFb receptors 1/2 (TGFBR1/2) and BMP receptors 1/2 (BMPR1/2; Weiss and Attisano, 2013). Binding of the TGFb or BMP dimer to this receptor complex causes the phosphorylation by the constitutively active type II kinase and activation (autophosphorylation) of the type I receptor, and the subsequent phosphorylation of the intracellular SMAD effectors by the activated type I serine/threonine kinase (Figure 4). The eight SMAD proteins are functionally grouped in five receptor-activated or R-SMADs, the common mediator or co-SMAD (SMAD4), and the two inhibitory or I-SMADs (SMAD6/7; Feng and Derynck, 2005; Weiss and Attisano, 2013). R-SMADs 1, 5, and 8 associate with and are phosphorylated by BMPR1, whereas R-SMADs 2 and 3 are activated by TGFBR1. Phosphorylated R-SMADs undergo a conformational change and dissociate from the type I receptor; two R-SMADs then form a trimeric complex with SMAD4 that is translocated into the nucleus and acts as a transcriptional regulator in association with other TFs and co-activators or co-repressors (Feng and Derynck, 2005; Weiss and Attisano, 2013; Figure 4). Despite its relative simplicity, TGFb/BMP signaling is complicated by the formation of different heterotetrameric receptor complexes with distinct ligand binding affinities and the existence of a variety of extracellular, transmembrane and nuclear co-factors and co-receptors for this pathway, making the signaling outcome highly specific for the corresponding ligand, cell type and context (Weiss and Attisano, 2013). Apart from the type I/II receptor serine/threonine kinases, SMADs are also phosphorylated by other pathways such as MAPK, cyclin-dependent kinases, protein kinase C, Ca^2+^/calmodulin-dependent kinase II (CAMK2), casein kinases I (CSNK1) and AKT (Feng and Derynck, 2005). TGFb/BMP signaling likewise activates SMAD-independent pathways, including MAPK, PI3K/AKT and small GTPases (Weiss and Attisano, 2013; Katagiri and Watabe, 2016; Figure 4).

TGFb and BMPs have been implicated in a variety of developmental processes, including mDA neuron induction, specification, differentiation and survival (Hegarty et al., 2014; O'Keeffe et al., 2017). Despite the wealth of data regarding their actions *in vitro*, relatively little is known about their *in vivo* functions in these contexts, especially for BMPs. Signaling components for both pathways, such as TGFB2/3 and certain BMP ligands (BMP5-7), TGFBR2 and BMPR1/2 receptors, SMAD3 and SMAD1/5/8 effectors, are expressed close to or within the developing mDA domain and in mDA neurons [(Hegarty et al., 2014; O'Keeffe et al., 2017) and references therein]. Accordingly, double null and compound heterozygote/null mutant *Tgfb2*/*3* and *Bmp5*/*7*, or *En1-Tgfbr2* and *Nes-Smad1* cKO embryos displayed a defective differentiation and early loss of mDA neurons, suggesting some redundancy in their developmental functions [(Hegarty et al., 2014; O'Keeffe et al., 2017; Chleilat et al., 2018; Brodski et al., 2019) and references therein]. Due to the early embryonic or perinatal lethality of these mutant mice, most studies were limited to prenatal stages. Notably, heterozygote and null mutant mice for some TGFb/BMP pathway components showed only a peri-or postnatal loss of mDA neurons, indicating that this signaling pathway also plays an important role in the adult maintenance of these neurons.

Homozygote *Tgfb3^−/−^* and *Smad3^−/−^* as well as heterozygote *Tgfb2^+/−^* mutants and mice expressing a DN TGFBR2 lacking the kinase domain in mature striatal neurons (*Camk2-*DN*Tgfbr2* mice) displayed a subtle postnatal (*Tgfb2^+/−^*, *Smad3^−/−^*, and *Camk2-*DN*Tgfbr2*) or pronounced (40%–50%) perinatal (*Tgfb3^−/−^*) loss of SNc DA/mDA neurons, which in *Tgfb3^−/−^* mice was due to their increased apoptotic cell death (Andrews et al., 2006; Zhang et al., 2007; Tapia-González et al., 2011; Tesseur et al., 2017). Severe locomotor deficits and a strongly reduced striatal DA innervation (*Camk2-*DN*Tgfbr2*), reduced striatal DA contents and/or increased DA turnover and oxidative stress (*Tgfb2^+/−^* and *Smad3^−/−^*) were also detected in these mice (Andrews et al., 2006; Tapia-González et al., 2011; Tesseur et al., 2017). Null mutant mice for the SMAD coactivator homeodomain interacting protein kinase 2 (HIPK2) displayed similar phenotypes (Zhang et al., 2007). *Tgfb2^+/−^* mice did not show an increased sensitivity to MPTP-treatment, whereas homozygote *Smad3^−/−^* and heterozygote *Smad3^+/−^* mutants exhibited a gene dosage-dependent onset (earlier in homozygotes and later in heterozygotes) and progressive perinuclear accumulation of SNCA aggregates as well as reduced astrocyte numbers in the SNc (Andrews et al., 2006; Tapia-González et al., 2011). These data suggested that TGFB2/3 have a pro-survival function in mDA neurons during periods of ontogenic cell death, but are not sufficient for the protection of adult mDA neurons against neurotoxic insults, whereas the SMAD3 effector of this pathway appears to have a more fundamental role in mDA and particularly SNc DA neuroprotection. Moreover, there might be some functional redundancy of the three TGFb ligands released not only from mDA neurons in the SNc, but also from their target cells in the striatum (Haas et al., 2016). Conditional removal of TGFBR2 in maturing mDA neurons of *Dat*-*Tgfbr2* cKO mice, however, did not alter the survival of these neurons during the first postnatal month but severely affected their axon and dendrite growth and spine formation in the striatum and SNr (Luo et al., 2016). A compensatory increase of GABAergic SNr neurons and upregulation of *Tgfb1* in these cells, as well as a reduction of excitatory (glutamatergic) synaptic inputs on mDA dendrites in the SNr, were also noted in the *Dat*-*Tgfbr2* cKO mice. These mice were hyperactive and had an impaired reward learning, probably due to the impaired balance between excitatory (less) and inhibitory (more) dendritic synaptic inputs and reduced burst firing of the mutant mDA neurons (Luo et al., 2016). In contrast to the prenatal brain, *Tgfb1* appears to be the only ligand expressed at detectable levels in postnatal SNc DA neurons and GABAergic SNr neurons (Luo et al., 2016). *Dat*-*Tgfbr2* cKO left SNc DA neurons unresponsive to SMAD2-mediated TGFB1 signaling and downregulated *Tgfb1* expression in these cells, suggesting an autocrine TGFB1/TGFBR2 signaling loop in SNc DA neurons (Luo et al., 2016). These findings indicated that apart from a direct pro-survival function in the mDA neurons themselves, TGFb signaling is also implicated in synaptogenesis and the establishment of the proper mDA connectivity with their afferent inputs and efferent targets. In support of the latter, one recently identified direct or indirect target gene of TGFb signaling in mature mDA neurons was the member of the Na^+^/Ca^2+^-exchanger family, *Slc8a3* (Chleilat et al., 2020). SLC8A3 is expressed in the SNc and was strongly downregulated in the absence of *Tgfbr2*-mediated signaling (Chleilat et al., 2020). Conversely, treatment of mDA-related cell cultures with TGFb strongly increased the SLC8A3 protein levels, but only in mature neurons and not in immature neural precursors. SLC8A3 suppressed the oxidative stress and promoted the survival of the differentiating mDA neurons in these cultures (Chleilat et al., 2020).

The embryonic or perinatal lethality of most BMP null mutants precluded the analyses of postnatal BMP functions. Heterozygote *Bmp7^+/−^* mice exhibited increased locomotor activities but no deficits in mDA neuron numbers and axonal or dendritic innervation of the striatum or SNr (Chou et al., 2008b). These mice, however, showed an increased sensitivity to the neurotoxic effects of methamphetamine: this drug elicited a greater reduction in locomotor activities and mDA dendritic and axonal innervation of the SNr and striatum, respectively, in the heterozygote compared to wildtype animals (Chou et al., 2008b). Methamphetamine also reduced the nigral and striatal levels of *Bmp7* mRNA, although it remains unclear whether this was a consequence of the reduced mDA innervation in the treated animals (Chou et al., 2008b). Mice expressing a DN BMPR2 lacking the intracellular kinase domains from the *Th* locus (*Th-*DN*Bmpr2* mice) displayed strongly reduced TH^+^ fiber densities in the striatum and SNr, reduced TH^+^ neuron densities in the SNc and particularly in the VTA, decreased locomotor activities and increased susceptibility to apoptotic cell death in the SNc (Chou et al., 2008a). However, targeting of the *Th* locus in the *Th-*DN*Bmpr2* mice affected TH expression itself, indicating that they need to be taken with caution (Chou et al., 2008a). Despite the sparse data about adult BMP signaling mutants, this pathway appears to have a stronger neuroprotective action on the mDA axonal and dendritic projections than on the cell bodies themselves. In support of a similar function in humans, the expression of *BMP2* showed the highest correlation with five mDA-specific marker genes in the human SNc and *BMP2* transcript levels were downregulated in late-stage PD (Goulding et al., 2019), whereas the protein levels of the inhibitory SMAD6 appeared to be upregulated in the SNc of PD patients (Vitic et al., 2021).

#### 3.1.4. WNT/b-catenin signaling

The WNT/b-catenin pathway has taken center stage in recent years in the context of mDA neurodegeneration and PD because of its mDA neuroprotective capacities and interaction with several PARK proteins (Berwick and Harvey, 2014; Marchetti et al., 2020). The evolutionarily highly conserved 19 WNT proteins in mammals signal *via* three distinct modes, the “canonical” or WNT/b-catenin pathway (discussed here), and the “non-canonical” WNT/Ca^2+^ and planar cell polarity (PCP) pathways (Wiese et al., 2018). The details of palmitoylated WNT secretion and diffusion across the extracellular space remain elusive, but the lipid modification makes them hydrophobic and possibly cell membrane-tethered or incorporated into exosomal vesicles (Nusse and Clevers, 2017). Thus, WNT signaling most likely has only a short range of action (Nusse and Clevers, 2017). In the WNT/b-catenin pathway, the absence of a WNT ligand results in the binding of cytoplasmic b-catenin (CTNNB1) by a “destruction complex” formed by the scaffold protein AXIN1/2, the adenomatosis polyposis coli (APC) protein and the constitutively active serine–threonine kinases CSNK1A and glycogen synthase kinase 3b (GSK3B). CSNK1A and GSK3B sequentially phosphorylate b-catenin at its N-terminus and this, in turn, is recognized by the beta-transducin repeat containing E3 ubiquitin protein ligase (BTRC), leading to the continuous ubiquitination and proteasomal degradation of b-catenin (Figure 4). WNT ligand binding to the extracellular N-terminal cysteine-rich domain of the seven-pass transmembrane Frizzled receptors (FZD1-10) induces their dimerization with the single-pass transmembrane LDL receptor related protein (LRP5/6; Figure 4) and phosphorylation of the cytoplasmic tail of LRP. The AXIN scaffold is subsequently recruited to the phosphorylated LRP tail, whereas the cytoplasmic C-terminus of FZD is bound by the disheveled segment polarity protein (DVL1-3). This presumably acts as a platform for the relocalization of the “destruction complex” to the cell membrane and its inactivation. Newly synthesized and unphosphorylated b-catenin accumulates in the cytoplasm and translocates into the nucleus, where it associates with DNA-bound TFs of the lymphoid enhancer binding factor (LEF1)/T-cell factor (TCF) family to transactivate WNT target genes (Figure 4). In the absence of b-catenin, LEF1/TCFs are bound by transcriptional co-repressors of the enhancer of split/groucho (TLE) family inhibiting WNT target gene activation. Several extracellular and transmembrane antagonists or agonists of WNT/b-catenin signaling modulate this pathway. The two transmembrane proteins, WNT target genes and WNT inhibitors zinc and ring finger 3 (ZNRF3) and ring finger protein 43 (RNF43) are E3 ligases that ubiquitinate the cytoplasmic loops of the FZD receptors, causing their rapid endocytosis and lysosomal degradation. Secreted R-spondins (RSPO1-4) are potent agonists of this pathway, especially at low WNT doses, bound with high affinity by the N-terminal extracellular domain of the seven-pass transmembrane leucine rich repeat containing G protein-coupled receptors (LGR4-6; Figure 4). RSPO proteins also interact with ZNRF3/RNF43, leading to their internalization and lysosomal degradation, and thus stabilization of the WNT/FZD/LRP complexes at the plasma membrane (Figure 4). Equally relevant in this context are the secreted WNT antagonists of the dickkopf (DKK1-4) and secreted frizzled related protein (SFRP1-5) families. DKKs bind to LRP5/6, potentially disrupting their WNT-induced dimerization with FZD receptors and WNT pathway activation (Figure 4), whereas SFRPs bind directly to WNTs, thereby inhibiting their interaction with FZD receptors (Nusse and Clevers, 2017). Different cell- and context-specific ligand/receptor pairings and cross-talk between the three WNT signaling pathways, together with the fact that b-catenin is also a major component of adhesion junctions in epithelia, complicate the interpretation of WNT/b-catenin signaling outcomes (Nusse and Clevers, 2017).

During mammalian mDA neuron development, it is particularly the WNT1-mediated b-catenin pathway which, together with FGF8 and SHH, plays a pivotal role in the patterning of the mid−/hindbrain region, induction of the mDA domain, mDA fate specification and differentiation of VM progenitors into mDA precursors/neurons [(Arenas, 2014; Joksimovic and Awatramani, 2014; Wurst and Prakash, 2014) and references therein]. *Wnt1* expression and b-catenin/LEF1/TCF-mediated signaling subside toward late midgestation and are barely detectable in the perinatal and adult rodent VM (L'Episcopo et al., 2011b, 2014; Bodea and Blaess, 2015; Zhang et al., 2015; Nouri et al., 2020). Other WNT ligands expressed in the developing rodent VM, such as WNT5A, WNT2 and WNT7A, either signal *via* alternative pathways (WNT5A) or their transcription in the rodent VM also declines during mid-stage development (*Wnt2* and *Wnt7a*) [(Brodski et al., 2019) and references therein]. These particular *Wnt* expression patterns made a role of WNT/b-catenin signaling in the maintenance of mDA neurons less likely during late development and adulthood.

Indeed, mDA/SNc DA neuron numbers and striatal innervation were not affected in adult *Dat-Ctnnb1*, *Th-Lrp5* and *Th-Lrp6* cKO mice (Diaz-Ruiz et al., 2012; Dai et al., 2014), whereas (Dai et al., 2014) reported a ~ 25% reduction of SNc DA neurons and striatal innervation in *Th-Ctnnb1* cKO mice. These discrepancies might be due to the earlier inactivation of the *Ctnnb1* gene in the *Th-Ctnnb1* (the precise timepoint was not determined, but TH starts to be expressed around E10.5 in the murine VM) compared to the *Dat-Ctnnb1* (~E15) mutants (Diaz-Ruiz et al., 2012; Dai et al., 2014). However, *Th-Lrp5*, *Th-Lrp6* and *Th-Ctnnb1 cKO* mice apparently had a reduced sensitivity to chronic MPTP treatment (more SNc DA neurons survived these treatments), which was interpreted as a neuroprotective effect of WNT signaling depletion in mDA neurons (Dai et al., 2014). As discussed below, this stands against most of the data available in this context. *Dat-Ctnnb1* cKO mutants, by contrast, showed an impaired acquisition and retention of motor skills, reduced response to locomotor methamphetamine sensitization, and synaptic transmission deficits in the striatum (Diaz-Ruiz et al., 2012). Although these findings already suggested a fundamental role of WNT/b-catenin signaling in adult mDA synaptic plasticity and neurotransmission, more definitive proof for such a role was provided by the inducible *Dkk1* OE in the striatum (*Camk2-iDkk1* mice; Galli et al., 2014). In line with the expression of several *Wnt* ligands, *Fzd* receptors, *Lrp* co-receptors and *Sfrp*/*Dkk* inhibitors in the postnatal and adult striatum, *Camk2-iDkk1* mice did not display any striatal MSN/IN or DA innervation/content deficits despite an apparently inhibited WNT/b-catenin pathway in this region (Galli et al., 2014). These mice, however, showed signs of degenerating striatal mDA synapses, impaired motor learning and a reduced locomotor sensitization to amphetamine (Galli et al., 2014), thus strongly resembling the *Dat-Ctnnb1* phenotype. In both cases, WNT/b-catenin signaling is expected to be abrogated in mDA neurons themselves or at least in their striatal terminals.

The first hints that WNT/b-catenin signaling is affected during mDA neurodegeneration in PD models and has a neuroprotective role in this context came from the analyses of 6-OHDA-treated rats and MPTP-treated mice, extensively reviewed elsewhere (L'Episcopo et al., 2018b; Marchetti, 2018; Marchetti et al., 2020). Briefly, inhibition of WNT/b-catenin signaling in the VM of 6-OHDA-treated rats (indicated by increased DKK1 and phosphorylated (activated) GSK3B, and decreased b-catenin levels) correlated with the 6-OHDA-induced mDA neuron loss (Dun et al., 2012). The mDA neurodegeneration in these rats was aggravated by application of rhDKK1 protein, whereas pretreatment with LiCl, a potent inhibitor of GSK3B (Figure 4), abolished this effect (Dun et al., 2012). Acute MPTP treatment of mice, a model of transient MPTP-induced mDA neurodegeneration recovering at later times post-lesioning, produced proinflammatory and chemokine-derived signals that strongly induced *Wnt1* transcription in VM reactive astrocytes (L'Episcopo et al., 2011b). Although other components of this signaling pathway, such as FZD1, CTNNB1 and GSK3B (Figure 4), were initially downregulated (FZD1 and CTNNB1) or upregulated (phosphorylated/activated GSK3B), suggesting inhibition of WNT1 signaling, they were up-or downregulated, respectively, at later timepoints indicating activation of this pathway, and this time-course correlated with the recovery of mDA neuron numbers and improvement of locomotor behaviors in the acute MPTP model (L'Episcopo et al., 2011b). Notably, the WNT1/FZD1/CTNNB1 mDA neuroprotective cascade was found to be downregulated during aging, resulting in a compromised mDA neuron recovery in aged mice (L'Episcopo et al., 2014). Likewise, intra-or supranigral infusion of recombinant DKK1 in untreated mice mimicked the temporary decline of mDA neurons and WNT/b-catenin signaling as well as astrocytosis observed in the acute MPTP model, exacerbated the aging-induced reduction of mDA neuron numbers, and reversed the positive effects of heterologous neural stem cell (NSC) grafting into MPTP-treated mice (section 4.1.4; L'Episcopo et al., 2011a, 2018a). Compelling evidence for a deregulated WNT/b-catenin signaling pathway in PD-related mDA neurodegeneration was also provided by the analyses of *PARK* gene-encoded proteins, especially LRRK2, the parkin E3 ubiquitin protein ligase (PRKN/*PARK2*) and the VPS35 retromer complex component (VPS35/*PARK17*; Berwick and Harvey, 2014). LRRK2 was proposed as a key scaffold protein interacting with DVL and other components of the antagonistic WNT/PCP pathway, but also with the intracellular LRP6 domain, AXIN, GSK3B and CTNNB1, thereby regulating the cytosolic “destruction complex” and b-catenin stability in the cell [(Berwick et al., 2019) and references therein]. LRRK2 KO enhanced, whereas LRRK2 OE inhibited WNT/b-catenin signaling (Berwick et al., 2017). Notably, most pathogenic *LRRK2*/*PARK8* mutations in fPD repressed, but one protective *LRRK2*/*PARK8* mutation increased WNT/b-catenin signaling (Nixon-Abell et al., 2016; Berwick et al., 2017). PRKN was reported to ubiquitinate CTNNB1, targeting it for proteasomal degradation and thereby suppressing excessive WNT/b-catenin signaling in adult mDA neurons, which might be neuroprotective for these neurons (Rawal et al., 2009). VPS35 is a key component of the retromer complex required for the retrograde sorting and recycling of transmembrane cargo proteins, including wntless (WLS), from endosomes to the cell membrane and *trans*-Golgi network (Sargent and Moore, 2021). WLS is essential for WNT intracellular transport and release (Nusse and Clevers, 2017). The only known VPS35 mutation associated with PD negatively interfered with WNT1/b-catenin signaling and SNc DA neuron survival in aging humanized mice carrying this mutation, although a disruption of the endosomal recycling of WLS was not demonstrated in these mice [(Sargent and Moore, 2021) and references therein].

### 3.2. Transcription factors

#### 3.2.1. EN1/2

The two Engrailed homeodomain proteins EN1/2 were among the first TFs recognized to have a cell-autonomous pro-survival role in developing mDA neurons, by regulating anti−/pro-apoptotic pathways and mitochondrial homeostasis in these neurons [(Alavian et al., 2014; Rekaik et al., 2015b) and references therein]. The *En1*/*2* double mutant mice (perinatal lethal) displayed the most severe phenotype in this context, indicating that both EN proteins act redundantly (Sgado et al., 2006). EN1/2 continue to be expressed in adult mDA neurons and control their age-dependent survival in an *En* dose-dependent manner, leading to a progressive decline of particularly the SNc DA neurons and striatal DA contents/neurotransmission as well as appearance of locomotor deficits in their absence (Sgado et al., 2006; Sonnier et al., 2007; Sgado et al., 2008; Nordström et al., 2015). These phenotypes were already detected in heterozygote *En1^+/−^* mice after the sixth postnatal week, including anhedonic and depressive-like behaviors probably due to the degeneration of VTA DA neurons also seen in these mutants (Sonnier et al., 2007; Nordström et al., 2015). The slow postnatal apoptotic demise of the SNc DA neurons in the *En1^+/−^* mice was enhanced after additional neurotoxic insults (Alvarez-Fischer et al., 2011; Rekaik et al., 2015a; Chatterjee et al., 2019), and was preceded by the early postnatal degeneration of their nigrostriatal projections, starting at the mDA terminals in the striatum and retrogradely proceeding toward the mDA cell bodies in the SNc (Nordström et al., 2015). Underscoring the versatility of these homeodomain proteins, acting not only as nuclear TFs but also as cytoplasmic regulators of translation or even “secreted” proteins that can be released by the producing cell and taken up by other cells (Prochiantz and Di Nardo, 2015), several proteins and pathways downstream of the EN proteins were subsequently identified as potential neuroprotective targets. They include nuclear encoded mitochondrial complex I subunits (Alvarez-Fischer et al., 2011), autophagic proteins and the mammalian target of rapamycin (mTOR) pathway (Nordström et al., 2015; Ghosh et al., 2016), anti-apoptotic and DNA repair proteins (Rekaik et al., 2015a), as well as neurotrophin/MAPK signaling (Alavian et al., 2009) and L1 retrotransposons associated with aging (Blaudin de Thé et al., 2018). In contrast to many other PD animal models (Dawson et al., 2018), adult *En1^+/−^* mice thus recapitulate several typical pathophysiological features of PD (Blesa et al., 2022). These mice have therefore been proposed as the animal model with particularly high construct validity for the study of this neurodegenerative disease (Nordström et al., 2015), although early developmental deficits cannot be ruled out in the germline heterozygote *En1^+/−^* mice. Understanding the precise role of the EN1/2 proteins in adult and aging mDA neurons therefore awaits the generation of *En1*/*2* cKO mutants.

#### 3.2.2. FOXA1/2

The forkhead/winged-helix TF FOXA2 was first implicated in the age-related degeneration of mDA neurons and the corresponding motor deficits by the analyses of 18 months old heterozygous *Foxa2^+/−^* mice (Kittappa et al., 2007). The lack of one *Foxa2* allele affected mostly the SNc DA neurons in an asymmetric manner, a key feature of PD (Balestrino and Schapira, 2020; Blesa et al., 2022), in ~30% of these aging *Foxa2^+/−^* mice (Kittappa et al., 2007). Subsequent studies showed that both orthologues, FOXA1 and FOXA2, are expressed by essentially all mDA neurons in the adult mouse brain, and redundantly required for proper gait and feeding behavior and the maintenance of mDA- and SNc-specific marker expression [TH, DDC, VMAT2, DAT, PITX3, *Drd2*, the retinoic acid and dopamine-metabolizing enzyme ALDH1A1, and the G protein-activated inward rectifier K^+^ channel GIRK2 (KCNJ6)] in SNc DA neurons during late embryonic/early postnatal and adult stages (Stott et al., 2013; Pristera et al., 2015). Notably, FOXA1/2 were not required for the adult survival of these neurons, because signs of apoptotic cell death and neurodegeneration were not detected in the *Dat-Foxa1/2* cKO mice (Stott et al., 2013; Pristera et al., 2015). Based on another study showing the co-expression and physical interaction of FOXA2 and NURR1 in adult mouse mDA neurons (Oh et al., 2015), a reduced binding of NURR1 to the *Th* and *Ddc* promoters in the absence of FOXA1/2 was proposed as one explanation for the adult *Dat-Foxa1/2* cKO phenotypes (Stott et al., 2013). Adult (>8 months) inducible *Dat-Foxa1/2* cKO mice displayed additional functional (reduced DA neurotransmission in the dorsolateral striatum and burst firing of SNc DA neurons) and behavioral (reduced feeding but no locomotor deficiencies) phenotypes (Pristera et al., 2015). The apparent discrepancy between the initial report of an mDA neurodegenerative phenotype in germline heterozygote *Foxa2^+/−^* mice and no signs of such a phenotype in the *Dat-Foxa1/2* cKO animals was only resolved by analyses of aging (>86 weeks) inducible *Dat-Foxa1/2* cKO mice (Domanskyi et al., 2014). At this age, a clear loss of SNc DA neurons and striatal DA contents became apparent in these mice, which was preceded by a gradual decrease of ALDH1A1-expressing SNc DA neurons and TH, DDC and DAT protein levels in these neurons as well as a gradual worsening of their locomotor abilities (Domanskyi et al., 2014). Importantly, the persistent absence of an mDA phenotype in inducible single-mutant *Dat-Foxa2* cKO mice was probably due to a compensatory upregulation of *Foxa1* in these mice (Domanskyi et al., 2014). Altogether, the FOXA1/2 TFs maintain the mDA and particularly SNc DA phenotype in adult neurons, most likely by the direct (as activating TFs) or indirect (through regulation of chromatin accessibility) transcriptional control of several genes involved in mDA identity and DA metabolism, which become compromised during aging and possibly also in PD. In fact, an age-related decline of FOXA2 protein levels was reported in mice, and a downregulation of *FOXA1*/*2* and several putative FOXA targets, such as *ALDH1A1*, *DAT*, *DDC*, *EN1*, *NURR1*, and *TH*, was detected in the SNc of PD patients but not age-matched healthy controls (Domanskyi et al., 2014; Oh et al., 2015).

#### 3.2.3. LMX1A/B

LMX1A/B are two LIM homeodomain proteins widely implicated in prenatal mDA neuron development. Transcription of *Lmx1a*/*LMX1A* in the adult and aging rodent and human brain remains controversial: some authors showed that mouse *Lmx1a*/human LMX1A expression is gradually downregulated after birth and not detected in adult mDA neurons (Hoekstra et al., 2013; Laguna et al., 2015), whereas others reported that mouse *Lmx1a* continues to be transcribed, albeit at lower levels, in adult and aging mDA neurons (Zou et al., 2009; Doucet-Beaupre et al., 2016). However, consensus exists that mouse *Lmx1b*/human LMX1B expression persists in these neurons throughout adulthood and during aging (Laguna et al., 2015; Doucet-Beaupre et al., 2016), although LMX1B appeared to be downregulated in nigrostriatal mDA neurons from PD patients (Laguna et al., 2015). The late embryonic *Dat-Lmx1a/b* cKO led to motor, non-motor (olfaction) and cognitive deficits in adult and especially aged mice (Laguna et al., 2015; Doucet-Beaupre et al., 2016). These behavioral impairments were accompanied by an age-dependent and progressive loss of mDA neurons and fibers in the striatum, decreased striatal DA contents and altered synaptic morphology of DA nerve terminals in this brain region of the *Dat-Lmx1a/b* cKO mice (Laguna et al., 2015; Doucet-Beaupre et al., 2016). Several of these striatal defects were also observed in adult inducible *Dat-Lmx1a/b* and *Dat-Lmx1b*, but not *Dat-Lmx1a*, cKO mice (Laguna et al., 2015; Doucet-Beaupre et al., 2016), indicating that the loss of LMX1B expression in postmitotic and adult mDA neurons affected primarily their nerve terminals in the striatum. The phenotypic integrity of the inducible *Dat-Lmx1a* cKO mice would be consistent with a lack of *Dat-Lmx1a* transcription in adult and aging mDA neurons. The synaptic deficits in the *Dat-Lmx1a/b* and *Dat-Lmx1b* cKO mice were traced back to ALP impairments, leading to an age-dependent accumulation of autophagic-lysosomal vesicles and pathologic (phosphorylated) SNCA in the dystrophic terminals of *Dat-Lmx1a/b* double-mutant mDA neurons (Laguna et al., 2015; Doucet-Beaupre et al., 2016). LMX1B appeared to activate a number of genes involved in ALP dynamics; the lack of this TF with the concomitant striatal nerve terminal deficits was thus counteracted, at least in part, by the systemic injection of the mTOR pathway inhibitor and ALP activator rapamycin into young *Dat-Lmx1a/b* cKO mice (Laguna et al., 2015). *Dat-Lmx1a/b* cKO mDA neurons *in vitro* also showed a downregulation of mitochondrial genes, impaired mitochondrial respiration, increased oxidative stress and mitochondrial DNA damage as well as altered autophagy, which however, did not correlate with changes in ALP gene transcription (Doucet-Beaupre et al., 2016). This suggested that LMX1A/B regulate mitochondrial complex I/III/IV genes and TFs, including the key regulator nuclear respiratory factor 1 (NRF1). Notably, adeno-associated virus (AAV)-mediated OE of *Nrf1* in *Dat-Lmx1a/b* cKO mice rescued their mDA phenotypic and locomotor deficits (Doucet-Beaupre et al., 2016). The LMX1B TF thus appears to play a crucial role in the adult maintenance of murine mDA neurons, by regulating the ALP and/or mitochondrial homeostasis in these cells and consequently the synaptic integrity of their nigrostriatal projections.

#### 3.2.4. NEUROD6

The basic helix–loop–helix (bHLH) TF *Neurod6* (*Nex1*) was identified as one of the differentially expressed genes between mouse SNc and VTA, which is confined to a VTA DA neuron subset co-expressing the TF OTX2, the Ca^2+^-binding protein calbindin (CALB1; calbindin-D28k) and ALDH1A1 (Khan et al., 2017; Figure 2D). Other markers, such as the neuropeptide gastrin releasing peptide (GRP/bombesin; ~78% of the *Neurod6*^+^ cells), the vesicular glutamate transporter 2 (VGLUT2/SLC17A6; 12%) and the Ca^2+^-binding protein calretinin (CALB2; 54%) were expressed in subsets of the *Neurod6*^+^ VTA DA neurons (Kramer et al., 2018; Bimpisidis et al., 2019). The NEUROD6^+^ neurons are located in the ventromedial VTA (PIF, dorsal PN and PBP), and project mostly to the NAc medial shell and to a lesser extent to other parts of the NAc and lateral septum (LS) in adult mice (Khan et al., 2017; Kramer et al., 2018; Bimpisidis et al., 2019; Figures 2B,D). They also exhibited characteristic electrophysiological properties that are distinct from the SNc DA neurons (Kramer et al., 2018). To determine the function of the NEUROD6^+^ VTA DA population *in vivo*, (Bimpisidis et al., 2019) disrupted the DA signaling in these neurons. This resulted in an increased locomotion upon psychostimulant administration, whereas optogenetic stimulation of these cells induced a place preference behavior, indicating that the NEUROD6^+^ VTA DA neuron subset is involved in the regulation of reward-related behaviors (Bimpisidis et al., 2019). The onset of *Neurod6* transcription in postmitotic mDA neurons at late embryonic stages and its persistence during adulthood suggested an important role of this TF in the maintenance of the corresponding VTA DA neuron subset (Khan et al., 2017). Deletion of *Neurod6* in *Nex1^Cre/Cre^* mice led to the postnatal loss of approximately one third of these neurons, particularly in the ventromedial VTA, and consequently of the innervation of one target area of these neurons in the forebrain (intermediate region of the LS; Khan et al., 2017). This was due to their apoptotic cell death and distorted mitochondrial metabolism (Khan et al., 2017). NEUROD1, another member of this bHLH TF family expressed in proliferating mDA progenitors but also in maturing and adult mDA neurons, might partly compensate the requirement of *Neurod6* for the survival of the NEUROD6-dependent VTA DA subset (Khan et al., 2017). A caveat of this approach, however, was the potential inactivation of *Neurod6* in several other VM populations during development (Kramer et al., 2018). Most importantly, the VTA DA population expressing exclusively NEUROD6 (NEUROD6^+^ and GRP^−^) was less vulnerable to neurodegeneration after 6-OHDA treatment of mice, although the downstream targets of this TF conveying these neuroprotective properties remain unknown (Kramer et al., 2018).

#### 3.2.5. NURR1 (NR4A2)

NURR1, a TF belonging to the nuclear hormone receptors and member of the 4A subfamily lacking known ligands (thus “orphan”), has attracted considerable attention because of its involvement in the specification of the mDA neuron phenotype during development and its anti-inflammatory properties (Decressac et al., 2013; Dong et al., 2016). NURR1 is widely expressed in the adult rodent brain, including the SNc and VTA (Zetterström et al., 1996; Kummari et al., 2021). In the healthy human brain, NURR1 was detected in virtually all mDA neurons at young adult age, but NURR1 as well as TH expression declined significantly in these neurons during aging (Chu et al., 2002). In the PD brain, NURR1 and TH expression were further reduced only in those SNc DA neurons displaying an SNCA pathology (Chu et al., 2006; Moran et al., 2007). *NURR1* is so far the only mDA developmental gene with identified rare mutations in fPD and iPD cases [(Wellenbrock et al., 2003; Decressac et al., 2013) and references therein], and belongs to the immediate early genes whose transcription is rapidly induced upon a variety of stimuli (Dong et al., 2016). Homozygote *Nurr1^−/−^* mice die shortly after birth (Decressac et al., 2013), but heterozygote *Nurr1^+/−^* mice showed an increased vulnerability to mDA neurotoxic insults by MPTP or proteasome inhibition (Le et al., 1999; Pan et al., 2008), and an age-related mDA neurodegeneration (Jiang et al., 2005; Zhang et al., 2012). The inducible *Dat-Nurr1* cKO during late development or adulthood resulted in a progressive loss of TH^+^, DAT^+^ and VMAT2^+^ SNc and VTA DA neurons and their striatal projections, reduced striatal DA contents and locomotor impairments (Kadkhodaei et al., 2009, 2013). This was interpreted as a partial loss of the mDA phenotype in the *Nurr1* mutant cells, because DDC expression remained unaffected and apoptotic cell death was not detected in the *Dat-Nurr1* cKO mice (Kadkhodaei et al., 2009, 2013). Adult depletion of *Dat-Nurr1* also resulted in fragmented and varicose mDA dendrites and axons, thus recapitulating a feature of human PD (Kadkhodaei et al., 2013). Interestingly, the vast majority of mostly downregulated genes in adult mDA neurons from the inducible *Nurr1* cKO mice belonged to nuclear-encoded mitochondrial genes and particularly to those involved in oxidative phosphorylation (Kadkhodaei et al., 2013). Furthermore, NURR1 expression in VM microglia and astrocytes appeared to protect mDA neurons from inflammation-induced neurotoxicity by direct binding of NURR1 to phosphorylated nuclear factor kappa B subunit 1 (NFKB1), one master regulator of pro-inflammatory genes, and recruitment of a REST corepressor complex to the promoters of these genes (Saijo et al., 2009). Downregulation of NURR1 expression in the mouse VM exacerbated the mDA neuron loss after an inflammatory insult (bacterial lipopolysaccharide or mutant SNCA), and upregulated the expression of several pro-inflammatory genes in these mice (Saijo et al., 2009). Additional interaction partners of NURR1 for adult mDA neuron maintenance include other nuclear receptors, such as retinoid X receptors (RXRs), and the TF FOXA2 (Decressac et al., 2013; Oh et al., 2015). Apart from the direct neuroprotective functions of this TF, NURR1 appeared to be engaged in an indirect mutual negative feedback loop with nuclear SNCA: *Snca* OE in the rat VM resulted in the concomitant downregulation of several *Nurr1* target genes involved in mDA phenotypic specification and survival, including the GDNF receptor *Ret* (Decressac et al., 2012; Volakakis et al., 2015; Figure 4). Accordingly, transgenic mice overexpressing mutant *Snca*, which do not display any obvious mDA deficits on their own, and at the same time lacking one *Nurr1* allele (*Nurr1^+/−^*; so-called “2-hit” mice), displayed an enhanced age-dependent reduction of NURR1 protein levels and decreased mDA neuron numbers, increased neuroinflammation and SNCA aggregation, and L-DOPA-responsive locomotor impairments with reduced penetrance (Argyrofthalmidou et al., 2021). One possible mechanism for the negative regulation of NURR1 by wild-type and mutant SNCA was recently shown *in vitro*: SNCA promoted the GSK3B (a WNT/b-catenin pathway component, section 3.1.4)-mediated phosphorylation and subsequent proteasomal degradation of NURR1 (García-Yagüe et al., 2021). The latter results indicated that pathogenic processes in PD might also afflict the normal expression and function of crucial “developmental factors” required for the phenotypic stability and proper survival of the mDA neurons in the adult and aging VM.

#### 3.2.6. OTX2

The orthodenticle homeodomain protein OTX2 is one of the first TFs expressed in the emerging mammalian embryo, involved in the initial steps of vertebrate gastrulation and axis formation as well as the correct establishment of the mid-/hindbrain boundary (Acampora et al., 2005). OTX2 is expressed throughout the VM and in mDA progenitors during early embryonic development, but later restricted to a VTA DA neuron subset co-expressing CALB1 in the central VTA (PIF and part of PBP), and CALB1 and ALDH1A1 in the ventromedial VTA (PN, IF), of the mouse, primate and human brain (Chung et al., 2010; Di Salvio et al., 2010b; Oosterveen et al., 2021; Figure 2D). Notably, OTX2 expression is excluded from adult SNc DA neurons and only rarely detected in GIRK2^+^ dorsolateral VTA (PBP) DA neurons, which also express SOX6 and high levels of the glycosylated (active) form of DAT (Chung et al., 2010; Di Salvio et al., 2010b; Panman et al., 2014; Oosterveen et al., 2021; Figure 2D). Consistent with the late restriction of OTX2 to a VTA DA subset, but somewhat surprising given its widespread expression in mDA progenitors and the strong reduction of mDA neuron numbers in *En1-Otx2* cKO mice, the survival of these mutant mice and their nigrostriatal projections or locomotor abilities were not affected, probably because of compensatory mechanisms during embryonic development (Borgkvist et al., 2006; Chung et al., 2010). However, the mDA innervation and DA contents of mesocorticolimbic target areas, including the PFC and NAc, were strongly reduced and the amphetamine-induced psychomotor behavior was altered in adult *En1*-*Otx2* cKO mice (Borgkvist et al., 2006; Chung et al., 2010), suggesting a preferential vulnerability of the VTA DA subset in the absence of *Otx2*. Subsequent analyses of *Dat*-*Otx2* cKO mice revealed that OTX2 was not required for the survival of VTA DA neurons in the adult mouse brain, but for the maintenance of their subset-specific identity and especially for the repression of GIRK2 and DAT in these neurons (Di Salvio et al., 2010a). Furthermore, OTX2 conferred neuroprotective properties to VTA and SNc DA neurons expressing high levels of DAT (which is required for the uptake of the active metabolite of the neurotoxic drug MPTP) *in vitro* and *in vivo*, by negatively regulating DAT expression in these cells after *Otx2* OE (Chung et al., 2010; Di Salvio et al., 2010a; Oosterveen et al., 2021). Other targets of OTX2 with potential neuroprotective functions in VTA DA neurons, although not demonstrated *in vivo*, might be axon guidance molecules, different neuropeptides, and proteins involved in the ER stress response (Chung et al., 2010; Chabrat et al., 2017; Oosterveen et al., 2021). Although OTX2 appears to be expressed in adult and aging human mDA neurons (Kamath et al., 2022), a similar neuroprotective role of this TF in the human context remains to be established.

#### 3.2.7. PITX3

The paired-like homeodomain TF PITX3 belongs to a family with three members in vertebrates and widespread functions during embryonic development (Tran and Kioussi, 2021). In the mouse brain, PITX3 expression is restricted to postmitotic mDA precursors and mature mDA neurons during development and adulthood (Tran and Kioussi, 2021). Although the majority of the adult mDA neurons co-express PITX3, point mutations causing a loss-of-function in aphakia (*ak*) and eyeless (*eyl*) mice, or inactivation of this gene in *Pitx3^−/−^* mice, primarily affected the vSNc DA subset, whereas the dSNc and VTA DA subpopulations were relatively spared in these mutant mice [(Li et al., 2009; Tran and Kioussi, 2021) and references therein; (Rosemann et al., 2009; Luk et al., 2013; Le et al., 2015)]. Accordingly, the *Pitx3* mutants also displayed a strong reduction of dorsolateral striatal mDA innervation (the nigrostriatal target area) and DA contents as well as locomotor deficits. The selective protection from neurodegeneration of the dSNc and VTA DA subsets in the *Pitx3* mutant mice appeared to be due to the mutually exclusive expression of CALB1 and PITX3 in these neurons (Luk et al., 2013; Figure 2D). The CALB1-expressing mDA population was selectively spared in both the SNc and VTA of the *Pitx3* mutants and after subchronic MPTP treatment of wildtype mice (Luk et al., 2013). The correct specification of the mDA neuron identity in the remaining cells, except for the two putative PITX3 targets *Dat* and *Vmat2* (Hwang et al., 2009; Jacobs et al., 2009), was not affected in the *Pitx3* mutant mice during development and adulthood, strongly suggesting that PITX3 is required for the proper survival of an mDA neuron subset in the pre- and postnatal mouse brain. The reduction of the *Pitx3* gene dosage in heterozygous *Pitx3^ak/+^* mutants, although not affecting their survival under normal conditions, resulted in a 44% loss of the SNc DA but not VTA DA neurons after subchronic MPTP treatment (Luk et al., 2013), indicating that PITX3 also protects a vulnerable SNc DA subset against neurotoxic insults in the adult mouse VM. A definitive proof of the adult neuroprotective and/or pro-survival function of PITX3 stemmed from analyses of inducible *Dat*-*Pitx3* cKO mice, in which the floxed *Pitx3* allele was removed postnatally at 2 months of age (Wang et al., 2021). In these mice, the SNc but not VTA DA neurons progressively degenerated during aging, thus becoming evident only after more than 6 months of age (4 months after *Pitx3* cKO; Wang et al., 2021). This was accompanied by an earlier reduction of striatal mDA innervation and DA contents, and a gradual worsening of their locomotor abilities. Because typical markers for these neurons did not appear to be changed in the remaining SNc and VTA DA neurons during aging, the progressive loss of the SNc DA subset in the inducible *Dat*-*Pitx3* cKO mutants was most likely due to their apoptotic cell death (Wang et al., 2021). The authors, however, noted a progressive reduction in the expression levels of two direct PITX3 targets, ALDH1A1 (Jacobs et al., 2007) and BDNF (Peng et al., 2011), and the unrelated GDNF, as well as an accumulation of SNCA and increased microgliosis and astrocytosis in the aging mutant VM. Because the striatum appeared to be the first site of evident phenotypic deficits in the *Dat*-*Pitx3* cKO mice, these data suggested that the mutant vSNc DA neurons initially undergo a retrograde degeneration from their axon terminals, potentially due to a defective retrograde neurotrophic support and DA neurotransmission. Later, these neurons probably succumb to a hostile environment due to the missing local neurotrophic support by BDNF, an increased DA oxidative and proteostatic stress load (lack of ALDH1A1 and SNCA accumulation), and heightened inflammatory responses in the adult and aging mouse VM in the absence of *Pitx3*. PITX3 was also shown to be upregulated downstream of retrograde (NFKB1-mediated) GDNF signaling *via* the RET receptor and GFRa co-receptors (Peng et al., 2011; Figure 4). This, in turn, activated BDNF expression in the adult rodent SNc, suggesting that PITX3 might also participate in a neuroprotective feedforward mechanism in response to neuroinflammatory stimuli (Peng et al., 2011). Despite this apparently prominent role of PITX3 in murine vSNc DA survival and neuroprotection, a recent transcriptomic analysis of laser-microdissected human vSNc and dSNc DA neurons from healthy adults revealed that *PITX3* is only expressed at subthreshold levels in the entire human SNc DA population (Monzón-Sandoval et al., 2020). The significance of PITX3 for human SNc DA survival and neuroprotection thus remains to be determined.

#### 3.2.8. SOX6

The SRY-box TF SOX6 was originally identified in a microarray-based screen of upregulated genes in mDA neurons derived from mouse embryonic stem cells (ESCs) after forced expression of lineage-specific TFs in appropriate culture conditions (signaling environments; Panman et al., 2011). The expression of this TF was subsequently mapped in single-cell RNA sequencing and other studies of the adult mouse and human VM/mDA domain to a specific mDA neuron subset co-expressing in their majority ALDH1A1 but not CALB1 (Panman et al., 2014; Poulin et al., 2020; Oosterveen et al., 2021; Pereira Luppi et al., 2021; Figure 2D). This mDA subset was located mostly in the vSNc and exhibited a higher vulnerability to neurodegeneration in the PD brain and after neurotoxic insults. Accordingly, the SOX6^+^ vSNc DA neurons projected preferentially into the dorsolateral striatum and responded to locomotor stimuli but not to rewards (Panman et al., 2014; Pereira Luppi et al., 2021). Some SOX6^+^ mDA neurons were also detected in the PBP (VTA) and RRF, and appeared to innervate parts of the ventromedial striatum, especially NAc core and lateral shell (Panman et al., 2014; Oosterveen et al., 2021; Pereira Luppi et al., 2021; Figures 2B,D). Analyses of *Sox6^−/−^* embryos essentially confirmed these findings and revealed that SOX6 and OTX2 act as opposing early fate selectors for vSNc DA and VTA DA neurons, respectively (Panman et al., 2014). The *Dat-Sox6* cKO in postmitotic mDA neurons did not seem to affect the total numbers of these neurons, but resulted in a reduced mDA dendritic fiber density in the VM, a progressive loss of striatal mDA innervation and in reduced DA contents, particularly in the dorsolateral target area of the vSNc DA neurons (Panman et al., 2014). These findings indicated that apart from being an important vSNc DA fate-specifying TF during development, SOX6 is also required to maintain some of their subtype-specific characteristics during adulthood, and most likely confers an increased vulnerability to degeneration to these neurons by so far unknown mechanisms. Indeed, SOX6 protein levels were reduced in the remaining pigmented (neuromelanin-positive) SNc DA neurons from PD patients (Panman et al., 2014) and, correspondingly, the murine SOX6^+^ mDA population showed an enrichment of pathways involved in PD pathogenesis (Pereira Luppi et al., 2021). Moreover, the lentiviral OE of SOX6 in human ESC-derived mDA neurons provided these cells with SNc DA-like properties and increased their susceptibility to mitochondrial stressors, which was probably also due to a generally increased energy metabolic rate in these cells (Oosterveen et al., 2021). It thus remains to be seen whether SOX6 acts upstream of some of these pathways and is involved in their regulation. A recent high-coverage single-nuclei RNA sequencing and spatial transcriptomic study confirmed that a SOX6^+^ mDA neuron subset (co-expressing the angiotensin II receptor type 1) located in the human vSNc is the population showing the largest decline in postmortem PD brains and the highest association with PD risk genes (Kamath et al., 2022). As a cautionary tale, this group also revealed a CALB1^+^ mDA neuron subset located in the dSNc that is only found in the primate (human) brain and not in rodents and other species (Kamath et al., 2022). Species-specific differences, including the exclusive existence of other or additional mDA subpopulations in the human brain, will thus have to be considered more thoroughly in the future.

#### 3.2.9. Additional transcription and other factors

In addition to the TFs described above, several other transcription and metabolic factors have been implicated in the maintenance and proper survival of adult mDA neurons in recent years. Among these are the homeodomain TF PBX1 (Villaescusa et al., 2016), the mesoderm specific transcript MEST (PEG1, an alpha-beta hydrolase protein involved in lipid metabolism; Mesman et al., 2016), and the zinc finger TF and BAF chromatin remodeling complex subunit BCL11A (Tolve et al., 2021).

RNA sequencing and subsequent *in situ* localization analyses showed that PBX1 is expressed from midgestational stages onward in postmitotic mDA precursors and adult mDA neurons in the mouse, and in the SNc DA subset of the adult human brain (Villaescusa et al., 2016; Kamath et al., 2022). The loss of *Pbx1* led to a compensatory upregulation of the orthologue PBX3, but depletion of both proteins revealed the requirement of PBX1 for the differentiation of an mDA precursor subset into PITX3^+^ and TH^+^ mDA neurons and, through prevention of their apoptotic cell death, their subsequent survival in the developing mouse VM (Villaescusa et al., 2016). PITX3 and the basic leucine zipper TF NFE2L1, which regulates the oxidative stress response by activating cytoprotective genes, were identified as direct target genes of PBX1 (Villaescusa et al., 2016; Figure 4). Notably, the expression of PBX1 and its target NFE2L1 were strongly downregulated or even absent in the remaining neuromelanin-containing (dark pigmented) vSNc DA neurons from the aging PD brain, suggesting that the lack of these two proteins contributes to the reduced survival of these neurons in PD (Villaescusa et al., 2016).

*Mest* was identified in a meta-analysis of transcriptional profiling data from the developing murine VM as a highly expressed gene during early mDA neuron development, which is later downregulated in these neurons (Mesman et al., 2016). From late embryonic stages on and in the adult mouse brain, *Mest* transcription appeared to be restricted to an SNc DA subset. Ablation of the *Mest* gene in mice, which is a maternally imprinted gene, resulted in the progressive and mostly postnatal loss of mDA neurons, affecting 25% or 42%, respectively, of the SNc DA subset in three or 8 months old *Mest^−/−^* mice (Mesman et al., 2016). This was accompanied by a 50% reduction in striatal mDA innervation and evoked DA release as well as locomotor deficits of the adult *Mest^−/−^* mice (Mesman et al., 2016). The authors suggested that the loss of *Mest* and *Pitx3* affected complementary mDA populations in the adult mouse SNc, but additional data are required to support this hypothesis. MEST thus appears to have a primarily pro-survival function in adult mDA and particularly SNc DA neurons, but its mode of action and relevance in the human context remains unknown.

BCL11A was identified because of its restricted transcription in the adult mouse VM and subsequently shown to be expressed in approximately one third of all mDA neurons (Tolve et al., 2021). The BCL11A^+^ mDA neuron subset was located predominantly in the lateral and dorsal tiers of the SNc, lateral VTA (PBP and CLi) and RRF (Tolve et al., 2021; Figure 2D). These neurons project mostly to the LS, NAc ventral and lateral shell, OT and parts of the dorsomedial and caudal striatum (Tolve et al., 2021). BCL11A expression initiates at late midgestation in the murine VM in what appears to be a postmitotic mDA precursor/neuron subset. The *Dat-Bcl11a* cKO did not affect the differentiation or survival of the corresponding mDA neurons, but altered their positioning in the SNc and VTA, and impaired the ability of the cKO mice to learn a motor skill (Tolve et al., 2021). The BCL11A-expressing mDA neuron subset was more susceptible to degeneration upon human *SNCA* OE in the adult mouse SNc, and the *Dat-Bcl11a* cKO strongly increased their vulnerability to death under these conditions (Tolve et al., 2021). BCL11A expression also labeled a distinctive subset of human mDA neurons generated *in vitro* from hiPSCs, which apparently was more amenable to degeneration after a neurotoxic (rotenone) insult (Tolve et al., 2021). Although the molecular targets and mechanisms of BCL11A action in these contexts remain to be established, this TF thus characterizes a mDA neuron subset with a higher vulnerability to neurodegeneration under adverse (neurotoxic) conditions, but also appears to confer directly or indirectly certain neuroprotective traits to these cells.

## 4. Developmental factors promoting mDA neuron survival in the aging or lesioned brain

Apart from their endogenous roles discussed in section 3, the exogenous application of several “developmental factors” contributing to the proper maintenance of mDA neurons in the adult brain is also beneficial for mDA neuron survival and/or neuroprotection during aging or after neurotoxic insults. In view of the existing reviews on this topic for each of the factors discussed below, this section summarizes only the most important and recent findings in this regard.

### 4.1. Signaling pathways

Given their secretory nature and their potential to act over longer ranges, signaling factors are particularly amenable for external manipulation by systemic or local administration. However, their mode of action and thus effectiveness is restricted by the availability of the receiving side, i.e., the appropriate receptors must be expressed in the mDA neurons and the danger of negative side-effects or unwanted targets in other cells poses an additional challenge in this regard.

#### 4.1.1. FGF signaling

The supranigral infusion of rhFGF20 protein shortly before and after a full or partial intranigral 6-OHDA lesion in rats protected approximately half of the mDA neurons against the neurotoxin-induced cell death and the concomitant loss of striatal mDA innervation (Sleeman et al., 2012; Boshoff et al., 2018). The rhFGF20-mediated mDA neuroprotection *in vivo* also counteracted the appearance of pronounced motor symptoms in the lesioned animals, whereas the treatment with an FGFR antagonist (Figure 4) before and after 6-OHDA lesioning exacerbated the mDA neuron loss and motor deficits in these animals (Boshoff et al., 2018). A major caveat of this approach was the requirement of an intracerebral application of the rhFGF20 protein because of its inability to cross the BBB. Therefore, Niu et al. (2018) devised a focused ultrasound-based method to deliver liposomes containing a rhFGF20 protein with improved solubility over the BBB into the 6-OHDA-lesioned rat brain. These liposomes released the rhFGF20 protein over a prolonged period of time and were capable of ameliorating the SNc DA neuron loss, striatal DA content reduction and motor deficits in the 6-OHDA-lesioned animals (Niu et al., 2018). An alternative approach searched for drugs reported to increase *FGF20* gene transcription, which were able to cross the BBB and were not contraindicated for the use in PD patients (Fletcher et al., 2019). Two compounds were identified, the β-adrenergic receptor agonist salbutamol and the antithrombotic and anticoagulant triflusal, which increased the FGF20 protein levels either only in the striatum or also in the VM, respectively, and provided a modest (approximately 13%–14%) protection of the SNc DA neurons in the adult rat brain after a partial 6-OHDA lesion (Fletcher et al., 2019).

One of the first *in vivo* experiments testing the neuroprotective properties of different factors was the intraventricular administration of FGF2 into the MPTP-lesioned primate brain (Pearce et al., 1996). This approach, however, did not lead to any improvements in locomotor behavior and SNc DA neuron numbers or striatal innervation, but caused an overproliferative response of the choroid plexus and ependymal cells lining the lateral ventricles of the treated animals, resulting in the appearance of hydrocephalus and additional neurological symptoms at high FGF2 doses (Pearce et al., 1996). One reason for this failure was the low stability and short half-life of this protein and its inability to cross the BBB (Pearce et al., 1996; Zhu et al., 2015). Attachment of a polyethylene glycol (PEG) moiety to the N-terminus of rhFGF2 (PEGylated rhFGF2) increased its thermal stability while retaining its biological activity (Zhu et al., 2015). Intravenous application of the PEGylated rhFGF2 in 6-OHDA-treated rats led to a significant improvement of motor behaviors, striatal DA contents and SNc DA neuron numbers, as well as a reduction of reactive astrocytes in the SNc, compared to the native rhFGF2 protein (Zhu et al., 2015). These effects were detected even after a prolonged cessation of PEGylated rhFGF2 treatment, and the concentrations of this modified protein in the SNc and striatum were higher compared to the native rhFGF2, indicating an improved bioavailability and better delivery across the BBB (Zhu et al., 2015). Such approaches might thus be exploited in other preclinical tests as a valid strategy to deploy bulky and unstable neuroprotective agents (proteins) into the human brain.

#### 4.1.2. SHH signaling

The unilateral intrastriatal or supranigral injection of a lipid-modified rhSHH-N protein into 6-OHDA treated rats or MPTP-treated marmosets, respectively, were among the earliest *in vivo* trials to test a neuroprotective action of this molecule (Dass et al., 2002; Tsuboi and Shults, 2002). Both groups reported a dose-dependent (the lowest dose usually being more effective) and only modest improvement of locomotor abilities and mDA neuron numbers or striatal innervation in the rhSHH-N-treated animals. Moreover, repeated application or higher doses of rhSHH-N protein had either no or detrimental effects on these two parameters in both studies, suggesting that methodological [(Tsuboi and Shults, 2002) injected the rhSHH-N before and after lesioning, whereas (Dass et al., 2002) only injected it after lesioning] and/or homeostatic aspects within the nigrostriatal system (section 3.1.2) influenced their outcome and have to be considered in this type of experiments. Gene therapy, i.e., the persistent expression of neurotrophins encoded by viral or other vectors delivered into the nigrostriatal system, is another approach in this direction (Blits and Petry, 2016). Using recombinant adenoviruses encoding SHH-N or the GLI1 TF, the adenoviral vectors were injected unilaterally into the dorsal striatum of rats subjected to a unilateral 6-OHDA lesion 1 week later, and shown to be retrogradely transported and translated into the corresponding proteins in the SNc DA cell bodies (Hurtado-Lorenzo et al., 2004). With the unmentioned assumption that the same number of mDA neurons was infected in each case, the SHH-N- and GLI1-encoding adenoviruses protected approximately 20% of the SNc DA neurons from degeneration but had no protective effects on their striatal axon terminals, which were lost to a similar extent as in untreated controls (Hurtado-Lorenzo et al., 2004). Indeed, an adenovirus encoding the GDNF neurotrophin still had the strongest neuroprotective effect on the SNc DA neurons and their striatal projections in this experimental setup (Hurtado-Lorenzo et al., 2004), an observation confirmed by another study (Tsuboi and Shults, 2002). Unfortunately, Hurtado-Lorenzo et al. (2004) did not assess the long-term and behavioral effects of their adenovirus-based treatments with SHH-N and its downstream TF GLI1 in neurotoxin-lesioned rats. Improvements in locomotor behavior as well as mDA neuron numbers and striatal DA content, turnover or innervation were detected after the intrastriatal delivery of SHH-N by an AAV vector followed by partial 6-OHDA lesioning of the rats 3 weeks later (Dass et al., 2005), or after 6-OHDA lesioning followed by intrastriatal delivery of a lentiviral vector encoding SHH-N (Zhang et al., 2014). The beneficial effects of these treatments appeared to be dependent on the concentration and a delayed action of SHH-N in the treated brains, because higher viral titers did not provide any mDA neuroprotection or locomotor improvements, and the latter appeared only 1 or 2 weeks post-lesioning (Dass et al., 2005; Zhang et al., 2014). Finally, systemic administration of the SHH agonist purmorphamine before MPTP-treatment of mice also had neuroprotective and anti-inflammatory effects in the SNc, which apparently were due to the SHH signaling-mediated activation of the PI3K/AKT pathway in this region (Shao et al., 2017; Figure 4).

#### 4.1.3. TGFbeta/BMP signaling

AAV-mediated OE of a constitutively active TGFBR1 (activating TGFb signaling in a ligand-independent manner) in the murine SNc prior to MPTP lesioning suppressed neuroinflammation in this region and rescued the degeneration of SNc DA neurons and locomotor deficits in these mice (Tesseur et al., 2017). Although 4 weeks old *Tgfb1^−/−^* mice did not show a mDA phenotype (Zhang et al., 2007), intracerebroventricular injection of TGFB1 protein 2 weeks after unilateral striatal MPTP lesioning inhibited the activation of microglia and release of pro-inflammatory cytokines in the SNc, and improved significantly the surviving mDA neuron numbers, striatal DA contents and locomotor behaviors of the treated rats (Chen et al., 2017). Subsequent *in vitro* experiments revealed that TGFB1-mediated signaling had an indirect neuroprotective action on mDA neurons by modulating the pro-inflammatory response and activation of VM microglia (Chen et al., 2017). Furthermore, MPTP lesioning downregulated the expression of TGFB1 and TGFBR1/2 as well as SMAD3 phosphorylation in the SNc, and TGFB1 treatment was able to counteract these effects (Chen et al., 2017).

Intranigral injection of BMP7 protein immediately before 6-OHDA lesioning had a dose-dependent (the lower dose being more effective) neuroprotective action on the ipsilateral mDA neurons and striatal projections, and reduced the motor deficits of the lesioned rats (Harvey et al., 2004). Intracerebroventricular injection of BMP7 also protected the treated mice from methamphetamine-induced bradykinesia and degeneration of the striatal mDA terminals (Chou et al., 2008b). Intracerebroventricular application of BMP7 after 6-OHDA lesioning did not rescue the locomotor deficits, but increased SNc DA neuron densities and striatal DA contents on both the ipsi-and contralateral sides of the lesioned rats (Zuch et al., 2004). Notably, lentiviral OE of BMP5/7 in the striatum led to a complete protection of mDA cell bodies and striatal innervation from degeneration, ameliorated the locomotor deficits and prevented the appearance of activated microglia and reactive astrocytes as well as the accumulation of aggregated SNCA in the SNc after simultaneous intranigral injection of mutant SNCA (Vitic et al., 2021). Altogether, the findings in neurotoxin-based animal models suggest that TGFb signaling might have a prevalent anti-inflammatory effect, whereas BMP signaling might have a predominant protective and restorative action on particularly the mDA neurons and their striatal projections.

#### 4.1.4. WNT/b-catenin signaling

Activation of WNT1/b-catenin signaling in the aging or lesioned rodent VM has potent neuroprotective and regenerative effects on mDA neurons *in vivo* (L'Episcopo et al., 2018b; Marchetti, 2018; Marchetti et al., 2020). Persistent and ectopic expression of *Wnt1* from one *En1* allele in *En1^+/Wnt1^* mice impeded the postnatal and progressive apoptotic cell death of mDA neurons normally observed in these *En1^+/−^* heterozygote mice (section 3.2.1; Zhang et al., 2015). The ectopic WNT1-mediated signaling strongly increased the transcript levels of the nuclear effector and direct WNT/b-catenin target gene *Lef1* in neighboring (most likely glial) cells to the mDA neurons (Zhang et al., 2015). This, in turn, moderately activated the transcription of the direct WNT/b-catenin and LEF1-bound target genes *Lmx1a*, *Dkk3* and *Fgf20*, and the indirect targets *Pitx3* and *Bdnf* [most likely *via* an activating LMX1A-PITX3-BDNF developmental gene cascade, (Arenas et al., 2015)] in the *En1^+/Wnt1^* VM (Zhang et al., 2015; Figure 4). Paracrine or autocrine action of the known mDA neurotrophin BDNF and the apparent pro-survival factor DKK3 (potentially signaling *via* alternative pathways such as TGFb/BMP, Figure 4) on the *En1* mutant mDA neurons sustained their survival and prevented their apoptotic cell death *in vitro*, despite the reduced *En1* transcript levels (at the expected 50%) in the heterozygote *En1^+/Wnt1^* mice (Zhang et al., 2015). Notably, FGF20 (section 4.1.1) did not prevent the demise of the *En1^+/−^* heterozygote mDA neurons in this paradigm (Zhang et al., 2015). Furthermore, the systemic application of a GSK3B inhibitor/WNT/b-catenin agonist (Figure 4), supranigral infusion of WNT1 protein or intranigral grafting of heterologous NSCs before or after MPTP lesioning counteracted the impaired WNT1/FZD1/CTNNB1 neuroprotective signaling and compromised mDA neuron recovery in aging and MPTP-treated mice (section 3.1.4.; L'Episcopo et al., 2011b, 2014, 2018a). Interestingly, activation of the same WNT1/b-catenin-induced *Lef1*-*Lmx1a*/*Dkk3*/*Fgf20*-*Pitx3*-*Bdnf* neuroprotective gene cascade mentioned above was detected in the MPTP-treated and NSC-grafted mice (L'Episcopo et al., 2018a; Figure 4). The systemic application of a GSK3B inhibitor before intranigral infusion of recombinant DKK1 in untreated mice or acute MPTP treatment also rescued the reduction of mDA neuron numbers observed in these two animal models (L'Episcopo et al., 2011a). Ectopic WNT1/b-catenin signaling in the adult and genetically compromised or lesioned VM thus has the capability of sustaining and eventually reactivating a neuroprotective gene cascade driven by this pathway during development, which is normally shut down due to the lack of *Wnt1* expression and WNT/b-catenin signaling activation in the unaffected adult and aging VM (section 3.1.4).

### 4.2. Transcription factors

Transcription factors act mostly as cell-restricted and nuclear instructive cues, in several instances immediately up- or downstream of the signaling cascades discussed above. Delivery of these factors into the VM or mDA neurons poses a difficulty as it usually implies genetic manipulations, for example, by local delivery of viruses or other types of transfection and genetic engineering, such as CRISPR/Cas-mediated gene activation. However, due to their activator or repressor function, TFs and their immediate or secondary targets may offer the advantage of a cell-specific mode of action triggering directly the neuroprotective and/or pro-survival cascades in the desired target cell(s).

#### 4.2.1. EN1/2

Homeodomain proteins, such as EN and OTX2, are exceptional in that they do not only work as nuclear TFs, but also as cytoplasmic regulators of protein translation and intercellular mediators that are released and taken up by other cells to control morphogenesis and physiological processes (Prochiantz and Di Nardo, 2015). Supranigral chronic infusions either alone or before lesioning, and acute injections after lesioning, of recombinant EN2, EN1 or mixtures of both proteins protected mDA (particularly SNc DA) neurons from degeneration in >6 weeks old *En1^+/−^* mice (Sonnier et al., 2007), subchronically MPTP-treated macaques (Thomasson et al., 2019), and mice treated subchronically with MPTP, intrastriatally or intranigrally with 6-OHDA, or receiving an intranigral injection of mutant SNCA (Alvarez-Fischer et al., 2011). The EN infusions rescued around half of the mDA neurons and led to significant improvements in locomotor behaviors of the treated mice. In the acute intranigral 6-OHDA mouse model, single injections of EN2 or OTX2 proteins potently prevented oxidative stress-induced DNA damage and chromatin remodeling as well as apoptosis in the mDA neurons, but these proteins had to be deployed within 24 h after lesioning to be effective (Rekaik et al., 2015a).

#### 4.2.2. FOXA2

In line with a functional cooperativity between FOXA2 and NURR1 (section 3.2.2), the combined uni- or bilateral injection of recombinant AAV vectors encoding these two TFs into the adult mouse VM, prior to subchronic MPTP lesioning, protected up to ~70% of the mDA neurons from death and led to significant improvements in locomotor behaviors as well as preservation of the striatal mDA innervation in these mice, whereas injections of single TF-encoding AAVs were much less effective (Oh et al., 2015). The AAV-mediated OE of the two transgenes (FOXA2 and NURR1) in the injected VM lasted for at least 6 months, and the neuroprotective effects persisted over 1 year under this regimen (Oh et al., 2015). A similar but delayed neuroprotective effect on mDA neurons, which lasted over the next 2 months, was observed when the two viruses were injected 2 days after the start of subchronic MPTP treatment (Oh et al., 2015). The unilateral injection of FOXA2 and NURR1 into the VM induced the expression of neurotrophic factors (BDNF, SHH, and TGFb), prevented the appearance of reactive astrocytes and microglia, and suppressed the transcription of proinflammatory cytokines on the injected side (Oh et al., 2015). These observations suggested that the mDA pro-survival effect of these two TFs in a lesion model of PD is mediated, at least in part, by the transcriptional activation (or suppression) and release (or not) of the previous factors either in the mDA neurons themselves or in surrounding non-neuronal cells (e.g., glia), thus generating an overall neuroprotective environment in the VM of the treated animals (Oh et al., 2015).

#### 4.2.3. NURR1 (NR4A2)

NURR1 is probably the mDA-associated TF that has been explored most intensively in preclinical trials for mDA neuroprotection in PD, based on its ability to directly activate several genes involved in mDA identity and metabolism, and at the same time to convey anti-inflammatory properties to the rodent VM (Decressac et al., 2013; Dong et al., 2016). Gene therapeutic approaches have shown that, consistent with the cooperative requirement of NURR1 and FOXA2 for mDA neuroprotection against age- or toxin-induced insults, intranigral AAV-mediated OE of these two TFs before lesioning promoted the survival of mDA neurons cell-autonomously (in these neurons themselves) and non-autonomously (*via* the surrounding glial cells) after subchronic MPTP treatment of mice (Oh et al., 2015; section 4.2.2). Notably, AAV-mediated OE of NURR1 alone was not or only marginally protective for mDA neurons in this PD model or after striatonigral retrograde transport in the unilaterally 6-OHDA-lesioned rat (Hurtado-Lorenzo et al., 2004; Oh et al., 2015). The requirement of NURR1 for adult mDA neuron survival also propelled the search for ligands and interaction partners of this orphan nuclear receptor as well as modulators of its expression in the VM, capable of rescuing at least some of the mDA deficits elicited by neurotoxic insults [(Dong et al., 2016) and references therein]. Among these were ligands for the heterodimer partner of NURR1, RXR, with either no [bexarotene, (Volakakis et al., 2015)] or good [the synthetic compound BRF110, (Spathis et al., 2017)] mDA neuroprotective effects *in vivo*; antimalarial drugs sharing a chloroquinoline moiety that directly bound to the ligand-binding domain of NURR1 and provided a remarkable mDA neuroprotection, anti-inflammatory response and behavioral improvements in 6-OHDA-treated rats (Kim et al., 2015); and the potential endogenous NURR1 ligands, prostaglandin E1 and its dehydrated metabolite PGA1, which improved the locomotor performance and mDA neuron survival in acutely or subchronically MPTP-treated mice (Rajan et al., 2020). Single or chronic treatments of MPTP- or 6-OHDA-lesioned mice with the synthetic RXR ligand BRF110 also improved their motor coordination abilities and did not lead to the appearance of dyskinesias in these mice, similar to the chloroquinolines but in contrast to L-DOPA treatment, suggesting that these drugs might also provide an improved symptomatic relief of motor impairments in manifest PD patients (Kim et al., 2015; Spathis et al., 2017).

#### 4.2.4. OTX2

OTX2 OE in SNc and VTA DA neurons that normally do not express this TF *in vivo*, or in primary VM cultures *in vitro*, conferred a dose-dependent and effective resistance against MPTP to these neurons, thus corresponding with its inherent neuroprotective function in a VTA DA neuron subset (Chung et al., 2010; Di Salvio et al., 2010a). Lentiviral OE of OTX2 in human ESC- and hiPSC-derived mDA neurons also provided these cells with VTA DA-like properties and protected them from rotenone- or MPTP-induced impairments in mitochondrial energy production and oxidative stress (Oosterveen et al., 2021). Single intranigral injections of OTX2 protein after an acute intranigral 6-OHDA lesion protected approximately half of the mDA neurons from their oxidative stress-induced death, but had to be deployed within 24 h after lesioning to be effective (Rekaik et al., 2015a; section 4.2.1).

#### 4.2.5. PITX3

Despite the strong evidences of a mDA pro-survival and/or neuroprotective function of PITX3 in the adult mouse brain (section 3.2.7), the AAV-mediated OE of PITX3 in the midbrain of healthy adult (3 months old) mice is one prime example of how such approaches can lead to undesirable outcomes (Kim et al., 2014). Although the ectopic PITX3 appeared to be mostly confined to the endogenous mDA (SNc and VTA) domain and did not change the numbers of mDA neurons and apoptotic cells in the AAV-infected VM up to 6 months later, a number of genes involved in DA biosynthesis, neurotransmission and mDA identity, as well as *Snca*, were upregulated in these mice (Kim et al., 2014). Furthermore, the striatal DA content and amphetamine-induced DA release were significantly increased, leading to an enhanced locomotor activity, reduced anxiety and improved motor learning of the PITX3-treated animals (Kim et al., 2014). Altogether, this suggested a hyperdopaminergic phenotype of these mice. Although the authors interpreted their findings as an indication that PITX3 protein levels must be tightly regulated in the mDA neurons, several technical details of their work remain to be clarified. Firstly, (Kim et al., 2014) used a FLAG-tagged version of PITX3, whose functionality and especially lack of interference with wildtype PITX3 and other TF-mediated gene activation or repression was not determined. This is particularly relevant because several genes were dysregulated in the PITX3-treated mice that are not established targets of this TF. Secondly, the authors did not show conclusively that PITX3 OE was restricted exclusively to mDA neurons and did not occur in other VM neuronal populations (e.g., SNr GABAergic neurons), whose altered excitatory or inhibitory output might interfere with normal mDA function. Thirdly, PITX3 OE in normally PITX3-negative mDA neurons (Luk et al., 2013) is also expected to alter the molecular make-up and function of these neurons in the context of the entire basal ganglia system, and might thus lead to abnormal behavioral outputs.

## 5. Discussion

Altogether, three major conclusions can be drawn from the findings summarized in the previous sections:

1. The extraordinary complexity of the pro-survival and neuroprotective networks for mDA and especially SNc DA neurons in the adult and aging mammalian brain, probably best exemplified by the four signaling pathways in this context (sections 3.1.1–3.1.4), make a more holistic view of the phenotypic changes after intrinsic or extrinsic manipulations of these networks imperative for future assessments of mDA neuron vulnerability and neuroprotection *in vivo* and, maybe even more importantly, *in vitro* [e.g., in human assembloid and organs-on-a-chip cultures (Reiner et al., 2021; Ingber, 2022)]. Such studies should not just focus on the mDA neurons in the VM and their forebrain projections, but even more on the functional interactions with their postsynaptic targets and presynaptic inputs in these brain regions. Indeed, some of the reported failures or unexpected outcomes in preclinical models of experimental PD treated with the previously identified neuroprotective agents *in vivo* (best exemplified by SHH signaling, section 4.1.2) might be directly related to the neglect of this complexity and the far-reaching interactions between mDA neurons and their efferent outputs as well as afferent inputs. Devising new preventive or neuroprotective and neurorestorative treatments for PD will therefore not be as simple as deploying a pro-survival/anti-apoptotic factor either systemically (e.g., into the blood stream) or locally (e.g., into the brain/VM or directly into the mDA neurons) to the patients in most cases. Details regarding the “site of action”-specific delivery, time-point(s) and mode of application as well as potential concentration-dependent interaction partners (i.e., side effects in the brain or body) of such a factor will have to be taken into consideration much more carefully in future approaches.
2. Apart from this intrinsic complexity of the mDA neuroprotective networks in the adult and aging brain, the notorious lack of a precise mechanistic knowledge of these networks (including all details of the signaling pathways and their specific downstream targets in this context) also hampers current approaches to preventive or disease-modifying therapies for PD. Much more and focused research in this direction is thus urgently needed to advance this field in the near future. Remarkably, germline and especially adult cKO mouse mutants for several of the signaling and TFs discussed in section 3 (*Shh*, *Tgfb*/*Bmp*, *Wnt*/*b-catenin*, *En1*/*2*, *Foxa1*/*2*, *Lmx1a*/*b*, *Nurr1*, *Pitx3*, and *Sox6*) display an adult-onset and progressive neurodegenerative process that resembles at least some of the early neuropathological features observed in human PD patients. These include a defective synaptogenesis and DA neurotransmission at their striatal terminals and dendrites in the SNr, the retrograde (striatonigral) degeneration of the mDA axons preceding the actual death of the mDA neurons, and an increased inflammatory response in the mutant VM. One striking observation in this context is that several of the “classical developmental TFs” for which direct target genes have been identified in the adult and aging brain, such as EN1/2, LMX1A/B and NURR1, appear to regulate the same pathways that are thought of primarily going awry in the PD brain, namely the mitochondrial energy metabolism, ALP and immune response/anti-inflammatory pathways. It is hard to imagine that these pathways and their target genes are not yet regulated in the mDA neurons in which these TFs are already active during prenatal and early postnatal development. Other “classical developmental TFs,” such as FOXA1/2, OTX2 and PITX3, appear to regulate mDA-specific target genes implicated in DA metabolism and neurotransmission, neurotrophic support and electrophysiology/Ca^2+^ homeostasis in the adult brain, which would have been the more expected outcome in this context. The latter targets are more related to the probably intrinsic metabolic stress load of the mDA neurons throughout their lifetime, discussed in section 2. It is thus very tempting to speculate that PD is ultimately a mDA-specific metabolic disease that might initiate already very early in life under adverse or disease-promoting conditions (mutations, excitotoxic or environmental insults, etc.), but remains undiscovered or “silent” over a considerable period of life due to the very slow kinetics of the molecular and cellular changes associated with the PD pathogenic process, and the remarkable ability of the mDA system to compensate these deficits over a significant time period. In view of the high conservation in mammals or even vertebrates of several “developmental” factors and signaling pathways as well as some of the *PARK* genes (Hernandez et al., 2016), the kinetics of the biochemical reactions controlled by these factors are expected to be very similar. This is particularly relevant for the preclinical modeling of this disease in animals, whose reliability, reproducibility and especially construct and predictive validity are anyway heavily debated in view of the many failures to translate neuroprotective approaches tested in these contexts into the human setting (Charvin et al., 2018; Dawson et al., 2018). As already proposed by others (Dawson et al., 2018), the average life expectancy of most of these PD animal models might be simply too short for these pathogenic metabolic processes to develop into the full phenotypes and symptoms associated with human PD. Emphasizing further the urgent need of “early” diagnostic biomarkers for this disease, and of improved human *in vitro* models for the entire mDA/basal ganglia system to understand its normal or aberrant function, respectively, in the healthy or PD brain. Lastly, these observations provide further support of a much earlier initiation of the PD pathogenic process in the human brain than currently anticipated, which would require a corresponding timely adaptation of any preventive or disease-modifying measures against PD (Figure 3B).
3. Directly related to the previous conclusion, there is also a remarkable paucity of knowledge about the “developmental physiology” of the mDA neurons. Almost nothing is known about the electrophysiological activities and Ca^2+^ homeostatic processes in the emerging mDA neurons, most likely playing a role already during the earliest stages of mDA neuron development and long before they have established a proper connectivity with their efferent and afferent targets, although their mitochondrial energy metabolism and autophagic/lysosomal clearance of misfolded proteins and defective organelles are now beginning to be unraveled in the human context *in vitro* (section 3.1.2). This fact has probably led to a collective neglect of these aspects arguing that they might not be biologically relevant, although several evidences have meanwhile accumulated suggesting the opposite is true for the developing mammalian VM and mDA neurons, both in the wildtype and in the PD context (Chan et al., 2007; Rockhill et al., 2009; Ferrari et al., 2012; Ramirez-Latorre, 2012; Kim et al., 2020; Carola et al., 2021; Akrioti et al., 2022; Stern et al., 2022; Virdi et al., 2022). It has been suggested that such ion- and neurotransmitter-driven early (immature) activities in developing neurons represent “phenotypic checkpoints” integrating intrinsic TF-mediated (genetic) developmental pathways with extrinsic signals from the surrounding environment to ensure the overall correct establishment of a neuron’s identity and function in the broader context of the nervous system (Ben-Ari and Spitzer, 2010). Accordingly, such “phenotypic checkpoints” may provide time-restricted windows of opportunity to modify or reset faulty but otherwise irremediable genetic programs during development, which are inactivated at the transition to adulthood and thus highly relevant in the context of gene therapeutic approaches to neurological human disorders (Ben-Ari and Spitzer, 2010). It is therefore suggested that more emphasis should also be placed on this aspect of mDA neuron development and adult vulnerability to degeneration in future studies.

## Author contributions

NP: conceptualization, writing—original draft preparation, writing—review and editing, and funding acquisition.

## Funding

This work was funded by Deutsche Forschungsgemeinschaft (DFG), grant number PR 629/3-1 (Projektnummer 408031320) to NP.

## Conflict of interest

The author declares that the research was conducted in the absence of any commercial or financial relationships that could be construed as a potential conflict of interest.

## Publisher’s note

All claims expressed in this article are solely those of the authors and do not necessarily represent those of their affiliated organizations, or those of the publisher, the editors and the reviewers. Any product that may be evaluated in this article, or claim that may be made by its manufacturer, is not guaranteed or endorsed by the publisher.
